# Chrysene‐Based Blue Emitters

**DOI:** 10.1002/chem.202001808

**Published:** 2020-10-19

**Authors:** Marvin Nathusius, Barbara Ejlli, Frank Rominger, Jan Freudenberg, Uwe H. F. Bunz, Klaus Müllen

**Affiliations:** ^1^ Max Planck Institute for Polymer Research Ackermannweg 10 55128 Mainz Germany; ^2^ Organisch-Chemisches Institut Ruprecht-Karls-Universität Heidelberg Im Neuenheimer Feld 270 69120 Heidelberg Germany; ^3^ InnovationLab Speyerer Str. 4 69115 Heidelberg Germany

**Keywords:** 2D acenes, blue emission, polyaromatic hydrocarbons, solubility increase, twisted biaryls

## Abstract

Chrysene and its bisbenzannulated homologue, naphtho[2,3‐*c*]tetraphene, were synthesized through a PtCl_2_‐catalyzed cyclization of alkynes, which also furnished corresponding biaryls subsequent to a Glaser coupling reaction of the starting alkynes. The optoelectronic properties of 5,5′‐bichrysenyl and 6,6′‐binaphtho[2,3‐*c*]tetraphene were compared to their chrysene‐based “monomers”. Oxidative cyclodehydrogenations of bichrysenyl and its higher homologue towards large nanographenes were also investigated.

## Graphical abstract

**Blue emitters**: The synthesis of new, soluble, stable, blue‐emitting materials based on chrysene is reported. Chrysene and its bisbenzannulated homologue, naphtho[2,3‐*c*]tetraphene, were synthesized through a PtCl_2_‐catalyzed cyclization of alkynes followed by the synthesis of the corresponding soluble biaryls. PAHs were further synthesized starting from the biaryls, leading to PAHs with unknown edge‐type structure.

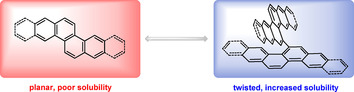

The development of new blue‐emitting materials based on polycyclic aromatic hydrocarbons (PAHs) continues to be an important challenge for organic chemistry and material science.^[1]^ During the last decades PAHs such as anthracene, phenanthrene, pyrene and corresponding biaryls with blue emission have been employed in optoelectronic devices such as OLEDs.^[2]^ A major advantage of PAH emitters is their increased stability.^[3]^ Chrysene is a well‐known PAH with blue fluorescence, but its derivatives are sparsely employed as emitting material in optoelectronic devices due to their low solubility.^[4]^ One possibility to address this issue is the substitution with solubility mediating groups such as aryl and alkynyl as well as (electron‐rich) amino substituents, reducing π–π stacking and aggregation.^[5]^ Along this line, several chrysene derivatives with high quantum yields were obtained.^[5]^ However, the HOMO–LUMO gap is affected by extension of the π‐system and/or by electron donating groups leading to red‐shifted emission bands. Similar to chrysene, dibenzochrysenes^[6]^ have attracted interest as they provide blue to blue‐green emission with remarkably high external efficiencies up to 2.0 % in OLEDs.[^7^, ^8^] However, among the different isomers, the synthesis of naphtho[2,3‐*c*]tetraphene is only reported in one article^[9]^ and due to its low solubility it remained poorly investigated. In this contribution, we report a new synthetic access to chrysene and its bisbenzannulated homologue as well as to their biaryl congeners to investigate their optoelectronic properties in terms of their applicability as blue emitters. Biaryl formation from strongly twisted subunits holds promise to retain the optical gaps without the use of functional groups. Further, the torsion between the two naphtho[2,3‐*c*]tetraphene or chrysene moieties increases solubility of the compounds by reducing π–π stacking.

Our synthetic access to chrysene and naphtho[2,3‐*c*]tetraphene as well as their homologues 5,5′‐bichrysenyl and 6,6′‐binaphtho[2,3‐*c*]tetraphene is depicted in Scheme 1. Key step in our synthesis was the Pt^II^‐catalyzed cyclization reaction of 1‐alkynylbiphenyls, either employing a terminal alkyne or a diyne to furnish π‐extended phenanthrenes. Suzuki coupling of 2‐naphthaleneboronic acid with ((2‐bromophenyl)ethynyl)trimethylsilane yielded **1** (93 %),^[5a]^ from which chrysene (**3**) was obtained in two steps through desilylation (92 %) and Pt^II^‐mediated cyclization as a colorless solid (81 %). 5,5′‐Bichrysenyl (**5**) was obtained from TMS‐protected **1** via Glaser coupling yielding **4** (67 %) followed by a selective twofold cyclization as a pale yellow solid (30 %).^[10]^ Treatment of **4** with trifluoromethanesulfonic acid in DCM^[11]^ instead of PtCl_2_ resulted in a partial rearrangement forming 6,6′‐bichrysenyl (see Supporting Information, Figure S45), inseparable from **5**. The π‐extended naphtho[2,3‐*c*]tetraphene and 6,6′‐binaphtho[2,3‐*c*]tetraphene were obtained similarly: Sonogashira coupling of 1,2‐bromoiodonaphthalene (**6**)^[12]^ with TMS‐acetylene furnished bromonaphthalene **7**, from which **9** was obtained via Suzuki reaction with anthracene‐2‐boronic acid (**8**) (54 %). Desilylation to alkyne **10** (67 %) followed by cyclization led to naphtho[2,3‐*c*]tetraphene (**11**) as a pale yellow solid in good yields of 80 %. This simple access to **11** significantly improved the hitherto reported synthesis involving the unstable 2‐methyl‐2*H*‐isoindole.^[9]^ **12** could not be obtained in a Glaser coupling reaction directly from the TMS‐protected alkyne **9** even at higher temperatures (up to 100 °C) and reaction times of up to 14 days. Using the more reactive terminal alkyne **10**, bialkyne **12** was isolated in yields of 57 % and subsequent PtCl_2_ induced cyclization led to 6,6′‐binaphtho[2,3‐*c*]tetraphene (**13**) as a yellow solid (87 %).

**Scheme 1:**
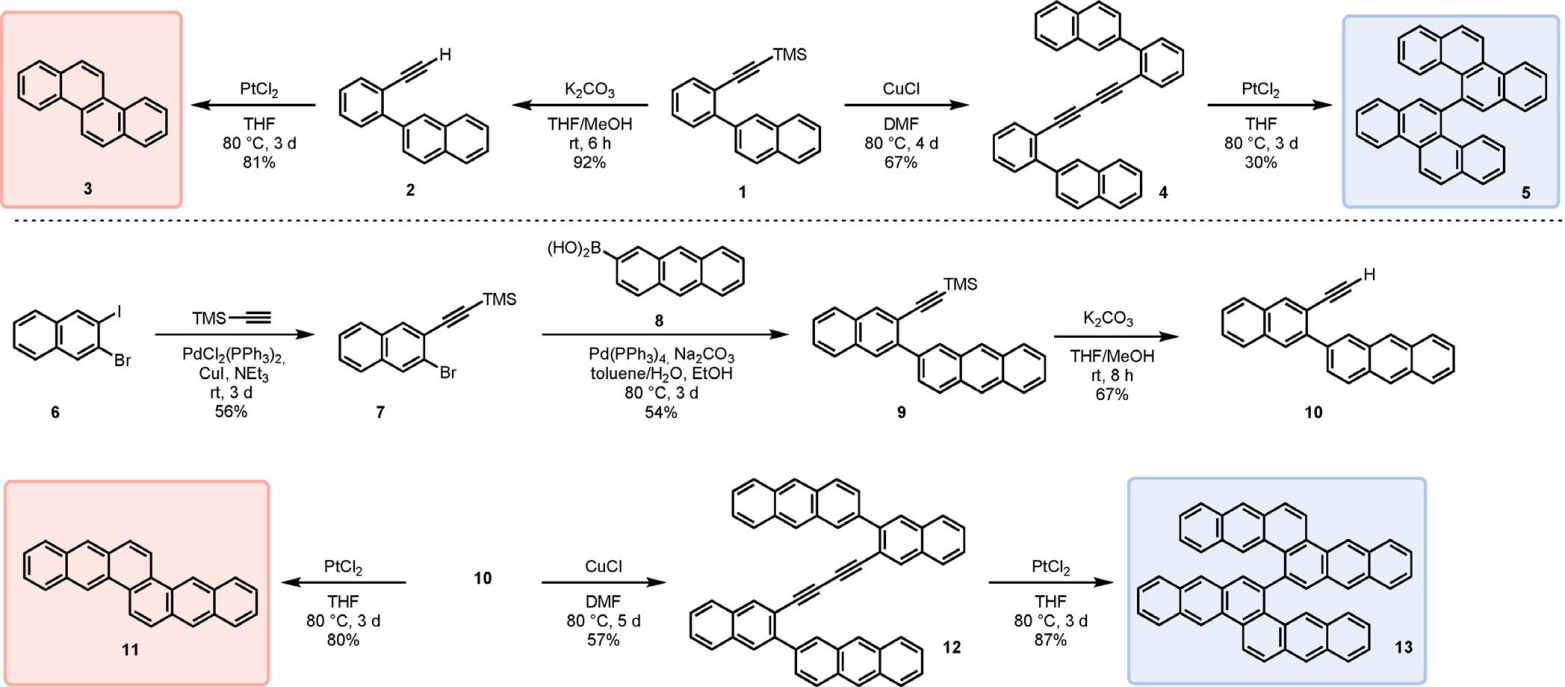
Synthesis of compounds **3**, **5** (top) and **11**, **13** (bottom).

Single crystals of **5** and **11** suitable for structure analysis were obtained after evaporation of a concentrated THF solution or slow cooling of a hot toluene solution, respectively. 5,5′‐Bichrysenyl (**5**) did not form π‐stacks—the chrysene moieties of neighboring molecules were oriented perpendicular to each other in an edge‐to‐face orientation. Due to intramolecular interactions, the chrysenyls adopted a torsional angle of ≈76° within the molecule (Figure 1, top), responsible for the dramatic increase in solubility: **5** (>10 mg mL^−1^ in DCM) was more soluble than chrysene (**3**) (4 mg mL^−1^ in DCM), which packs in a herringbone motif (see Supporting Information, Figure S13).^[13]^ Planar naphtho[2,3‐*c*]tetraphene (**11**) also crystallized in this motif (Figure 1, bottom).

**Figure 1:**
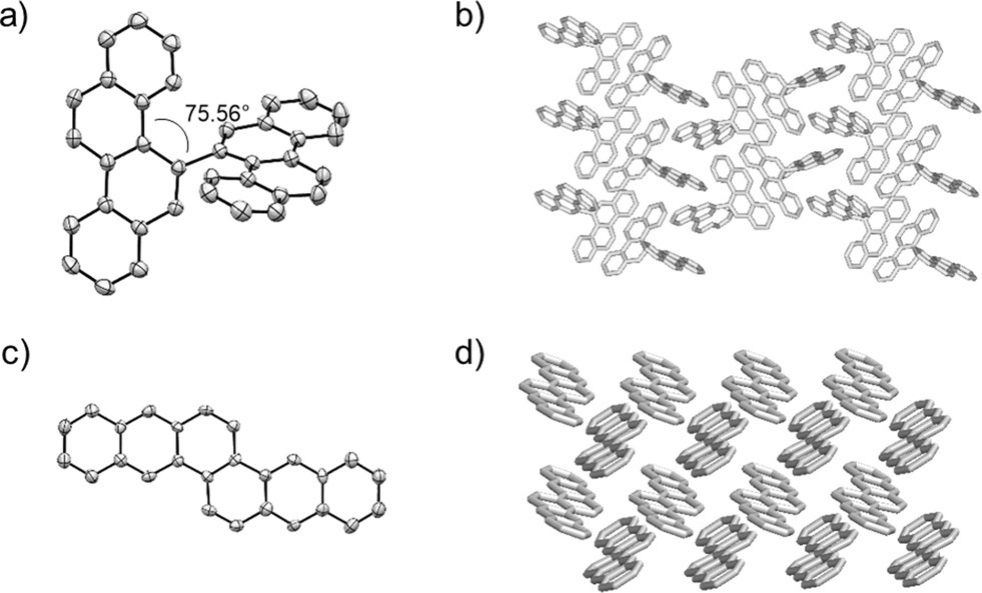
Crystal structure and packing of **5** (a, b) and **11** (c, d).

Compared to its monomer **11** (3 mg mL^−1^ in THF), the solubility of **13** nearly doubled (5 mg mL^−1^ in THF), for which unfortunately only amorphous solids were obtained after various crystallization attempts. Thus, as expected, formal homocoupling resulted in a remarkable solubility increase, even without the introduction of solubility mediating groups, which was rationalized in terms of the twisted nature of the biaryls.

We analyzed the absorption and photoluminescence of the target compounds in *n*‐hexane and in thin films (Figure 2). While the normalized absorption spectra of chrysene and 5,5′‐bichrysenyl were nearly identical in solution with absorption of the latter tailing into the visible region, the emission signal of 5,5′‐bichrysenyl was red‐shifted by 58 nm compared to purple‐blue emitting chrysene, leading to a sky‐blue emission. A similar shift was observed for **11** and **13**, the emission maxima changed from 421 nm to 472 nm furnishing a blue‐greenish emission. The quantum yield QY in solution (determined with an Ulbricht sphere) increased noticeably between chrysene and naphtho[2,3‐*c*]tetraphene from 11 % to 46 % and was slightly improved in the biaryls (16 % for 5,5′‐bichrysenyl and 49 % for 6,6′‐binaphtho[2,3‐*c*]tetraphene).

**Figure 2:**
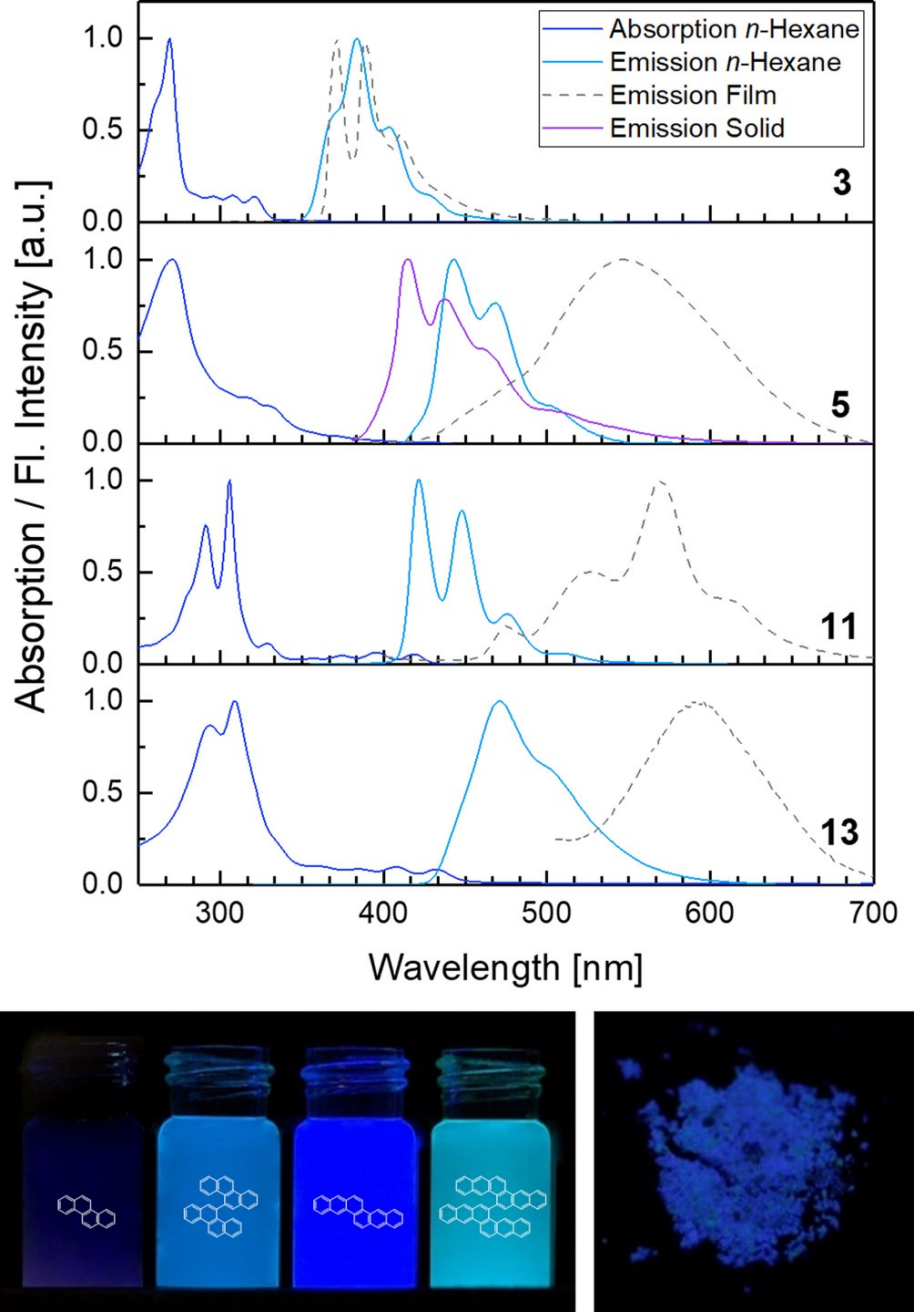
Top: Absorption (solution) and emission spectra (solution, film) of **3**, **5**, **11** and **13** in *n*‐hexane. *λ*_ex_ in solution: **3**: 330 nm, **5**, **11**, **13**: 345 nm, *λ*_ex_ in film: absorption maxima (see Supporting Information, Figure S7). Bottom: Photographs of solutions of **3**, **5**, **11** and **13** in *n*‐hexane (left) and crystalline **5** (right).

Stability measurements involving **5** and **13** revealed no change of absorption spectra over a period of 5 days under continuous irradiation with a 365 nm hand‐held UV lamp under aerobic conditions in solution (see Supporting Information, Figures S9 and S10), underlining the high photostability of the compounds.

Emission of thin films of **5**, **11** and **13**, spin‐cast from THF, was dramatically red‐shifted compared to that observed in solution: 5,5′‐bichrysenyl (**5**) displayed a yellow photoluminescence with a broad band around *λ*_max,film_=546 nm, bathochromically shifted by 104 nm, and naphtho[2,3‐*c*]tetraphene (**11**) and 6,6′‐binaphtho[2,3‐*c*]tetraphene (**13**) were yellow‐orange fluorescent in films, with red‐shifted maxima by 149 nm and 121 nm, respectively. Only the thin film emission spectrum of chrysene itself resembled its solution spectrum, although a slight change in the intensity distribution of its vibronic structure was observed. The otherwise red‐shifted emission bands indicated significant π–π interactions of the chromophores of **5**, **11** and **13** in thin films. Interestingly, crystalline 5,5′‐bichrysenyl (**5**) (*λ*_max,cryst_=415 nm) was blue emissive (see Figure 2, bottom right), blue‐shifted by 27 nm compared to emission in solution and by 131 nm compared to films. In silico analysis of the rotamers of 5,5′‐bichrysenyl by stepwise changing the torsional angle (DFT, B3LYP/6‐311++G**, gas phase, see Supporting Information Figure S15) yields a potential with a minimum at 78°, in accordance with the value obtained via X‐ray structure analysis. Between 30° and 150°, **5** rotates at room temperature as the potential energies do not exceed 60 kJ mol^−1^ until the aforementioned torsional angles are reached.^[14]^ As a consequence, different conformations and morphologies in kinetically trapped thin films compared to the single crystals are expected even at ambient temperatures. The frontier molecular orbitals were, even in the biaryl systems, located on the entire π‐system with a nodal plane on their single bonds. Biaryls **5** and **13** displayed only slightly increased gaps (*E*_g,calc_=3.73 eV/2.67 eV) compared to that of **3** and **11** (*E*_g,calc_=3.90 eV/2.75 eV), which are in good agreement with the optical gaps determined from their absorption onsets (see Table 1), due to the twisted structures. Cyclovoltammetry supports the calculated LUMO levels (see Supporting Information, Table S1). The dramatically red‐shifted photoluminescence in thin films was thus explicable by the wide range of torsional angles of the biaryl systems resulting in partial planarization and intermolecular (π–π) interactions. This feature also explains the red‐shifted emission of **5** in solution compared to its crystalline state as well as the red‐shifted emissions of biaryls **5** and **13** compared to their monoaryls **3** and **11** in solution. To retain blue emission and to reduce red‐shifts of these biaryls, both in thin films and in solution, partial planarization needs to be prohibited by steepening the torsional potential curves. This could either be achieved through *ortho*‐functionalization, for example, with methyl substituents, resulting in a reduction of the bandwidth of adoptable angles at room temperature (50° to 130°) or by going to terchrysene‐based systems. Additionally, alternative ways of film formation, other than spin‐coating, allowing for more time for the biaryl rotamers to equilibrate and adopt their energetically preferred (twisted) conformation should furnish blue‐emitting thin films for use in OLEDs.

**Table 1:** Summary of the optoelectronic and quantum chemical characterization of compounds **3**, **5**, **11**, 1**3**.

	*λ*_abs_^[a]^ [nm]	*λ*_em_^[a,b]^ [nm]	*λ*_abs_^[c]^ [nm]	*λ*_em_^[c]^ [nm]	*ϵ* [L mol^−1^ cm^−1^]	QY solution/film	*τ*_f_ [ns]	HOMO/LUMO^[d]^ [eV]	*λ_onset_* ^[f]^/gap calcd.^[d]^ [eV]
**3**	269	384	278	371	3.4×10^4^	0.11/0.03	9.7	−5.71/−1.81	3.75/3.90
**5**	271	442	278	546(415)^[e]^	5.4×10^4^	0.16/0.04	2.4, 9.6	−5.56/−1.83	3.56/3.73
**11**	306	421	274	570	5.6×10^4^	0.46/0.03	8.6	−5.19/−2.44	2.89/2.75
**13**	308	472	270	593	4.8×10^4^	0.49/0.05	2.7, 9.4	−5.13/−2.46	2.77/2.67

[a] All spectra were measured in *n*‐hexane. [b] Excitation wavelength: **3**: 330 nm, **5**, **11**, **13**: 345 nm. [c] Thin films were spin‐coated from THF, excitation wavelength: absorption maxima. [d] Frontier molecular orbital energies were obtained from quantum‐chemical calculations with Gaussian16 B3LYP/def2SVP//Gaussian 16 B3LYP/def2TZVP. [e] Emission maximum of 5,5′‐bichrysenyl in single crystals. [f] Absorption onset in *n*‐hexane.

The synthesis of large PAHs without solubility mediating groups is a challenge for solution‐based chemistry. Soluble biaryls, such as **5** and **13**, may serve as precursors for large, unsubstituted PAHs—this approach could thus complement the hitherto explored one via exhaustive cyclodehydrogenations of polyphenylene dendrimers which are planarized under Scholl conditions.^[15]^ With compounds **5** and **13** in hand, we further investigated the Scholl cyclodehydrogenations to obtain **14** and **15**, respectively, which, as a member of the larger PAHs might be utilized as IR emitters and/or be utilized for stimulated emission.^[16]^ Only one synthetic approach to **14** was reported so far but separation from a naphthoindenozethrene^[17]^ side product was only achieved in minuscule amounts by HPLC due to the low solubility, therefore a selective access is of interest. Bichrysenyl derivatives were employed by us to obtain several large PAH systems, such as graphene nanoribbons and (dibenzo)ovalenes.[^10a^, ^10b^, ^10c^] Based on our experience with Scholl reactions we tried three literature reported reaction conditions: DDQ/CF_3_SO_3_H, FeCl_3_ and DDQ/ScOTf_3_.[^10c^, ^18^] Only the combination of DDQ with trifluoromethanesulfonic acid furnished **14** as a deep red double [4]helicene (Scheme 2) with a maximum yield of 70 %, identified by HR‐MALDI (see Supporting Information, Figure S4). Its low solubility prohibited analysis by NMR spectroscopy, even at high temperature. The planarization caused a red‐shift of both absorption (*λ*_abs_=495 nm) and emission (*λ*_em_=516 nm) maxima—**14** is yellow fluorescent in *n*‐hexane. The observed small Stokes shift illustrated the rigidity of the compound. This is the first selective access to **14** in reasonable yields.

**Scheme 2:**
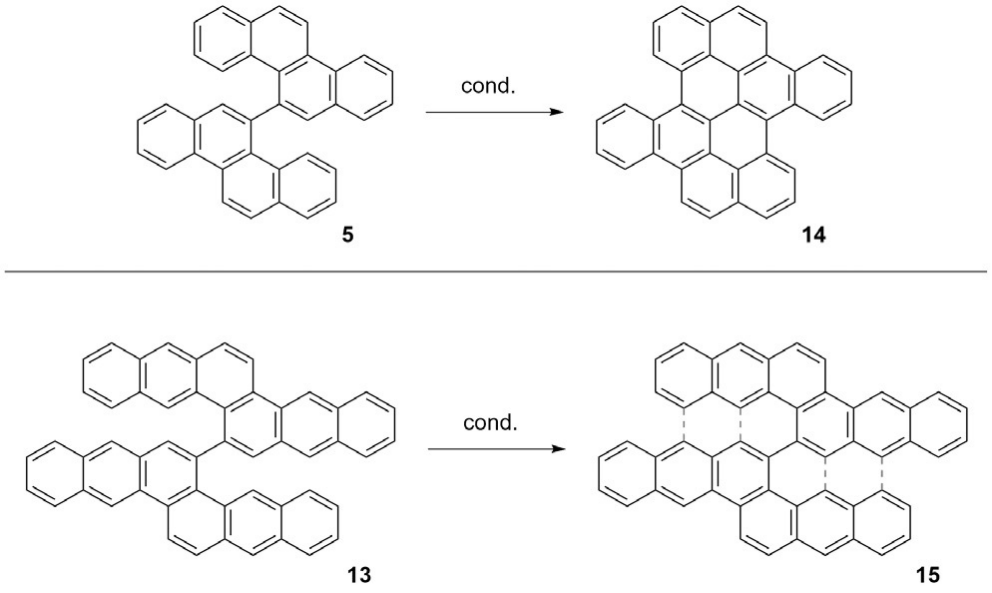
Cyclodehydrogenation reaction to **14** and **15**. Conditions: DDQ, CF_3_SO_3_H, DCM, −78 °C–rt, 2 h.

Cyclodehydrogenation of 6,6′‐binaphtho[2,3‐*c*]tetraphene (**13**) under similar conditions only lead to an insoluble and inseparable mixture of twofold cyclodehydrogenated product of unknown identity and, in traces, to fully oxidized **15** (see Supporting Information, Figure S5 for mass spectra). Higher temperatures and prolonged reaction times up to 7 days did not drive the reaction to completion. While the solubility of **11** as well as that of its monocyclodehydrogenated reaction intermediate is sufficiently high for a complete fusion to yield **14**, the limit of this approach is quickly met in terms of solubility of the reaction intermediates when attempting to synthesize **15**. As such, cyclodehydrogenation cannot compete with oligophenylene planarization in terms of PAH size,[^15a^, ^15b^] but offers an access to smaller derivatives with cove edges inaccessible via the latter strategy. Whether **11** really poses the limit to PAH structures via the biaryl approach in terms of size or only its bisbenzannelated derivative, for example, 13,13′‐bibenzo[*c*]tetraphene, may also be fully dehydrogenated, remains to be investigated.

In conclusion, we reported a simple PtCl_2_‐mediated synthetic strategy towards chrysene (**3**), 5,5′‐bichrysenyl (**5**), naphtho[2,3‐c]tetraphene (**11**) and 6,6′‐binaphtho[2,3‐*c*]tetraphene (**13**) via 6‐*endo*‐dig cyclizations of 1‐alkynylbiaryls and their Glaser coupled bialkynes. Although biaryl formation does only slightly influence the calculated frontier molecular orbital energy levels, it results in an increased solubility without additional solubility‐mediating groups. The shallow torsional potential, not greatly hindering rotation about the connecting single bond, leads to a pronounced red‐shift in the thin film emission due to partial planarization and intermolecular interactions. In the crystalline state, however, blue emission is retained due to the herringbone motif of the twisted biaryls. Key to soluble, all sp^2^ hybridized, blue fluorescent derivatives for OLED applications is thus to rigidify the system even further, for example, as in 5,11‐polychrysenylene, which also served as a graphene nanoribbon precursor.^[10a]^ A new approach through polymerization of dihalogenated bialkyne **4** has the potential to exceed the previously reported oligomers in size due to reduced steric hindrance upon C−C bond formation. Whereas polyphenylene dendrimers are conventionally employed as precursors to large unsubstituted PAHs, as they planarize under Scholl conditions, we explored a new strategy through cyclodehydrogenation of (soluble) biaryl systems in this work, which could furnish nanographenes with hitherto unexplored edge type combinations. These types of sparsely investigated PAHs are of interest for lasing applications^[19]^ and NIR emission.^[16]^ We investigated oxidative Scholl coupling of **11** furnishing **14**, but full cyclodehydrogenation of its higher homologue **13** was only observed in trace amounts: Most likely, solubility limits the full conversion of the twofold cyclodehydrogenated intermediate, which will be circumvented by utilizing surface‐assisted fusion^[20]^ in the future.

## Experimental Section

### Crystallographic data

Deposition numbers 1996744, 1996745, 1996746, 1996747, 1996748, and 1996749 contains the supplementary crystallographic data for this paper. These data are provided free of charge by the joint Cambridge Crystallographic Data Centre and Fachinformationszentrum Karlsruhe Access Structures service.

### Syntheses

**General procedure for the synthesis of acenes**: General procedure (GP): In a flame dried Schlenk flask, the starting material (1.00 equiv.) and PtCl_2_ (0.25 equiv.) were dried for 1 h under vacuum. Dry toluene was added and the reaction was stirred at 80 °C for 3 d. The reaction mixture was allowed to cool to room temperature and purified. The crude product was further purified by flash chromatography (SiO_2_, PE/EA 10:1) to obtain the compound.

**5,5′‐Bichrysenyl (5)**: According to GP, **4** (600 mg, 1.32 mmol, 1.00 equiv.) and PtCl_2_ (87.7 mg, 329 μmol, 0.25 equiv.) were dried for 1 h under vacuum. 20 mL of dry toluene (freeze pumped three times) were added and the reaction was stirred at 80 °C for 3 d. The mixture was allowed to cool to room temperature before the solvent was removed under reduced pressure. The crude product was purified by flash chromatography (SiO_2_, PE/EA 10:1) to obtain **5** as a pale yellow solid (180 mg, 1.32 mmol, 30 %). *R*_f_=0.29 (SiO_2_, petroleum ether, ethyl acetate 10:1, *v*/*v*), M.p.: >300 °C, ^1^H NMR (600 MHz, CD_2_Cl_2_, 295 K): *δ*=8.98 (d, *J=*9.16 Hz, 2 H), 8.94 (d, *J=*8.88 Hz, 2 H), 8.28 (d, *J=*9.09 Hz, 2 H), 8.16 (d, *J=*9.13 Hz, 2 H), 7.98 (d, *J=*8.01 Hz, 2 H), 7.80–7.74 (m, 4 H), 7.66 (s, 2 H), 7.62 (td, *J=*7.43, 0.92 Hz, 2 H), 7.37 (td, *J=*7.40, 0.99 Hz, 2 H), 6.88 ppm (td, *J=*7.85, 1.51 Hz, 2 H), ^13^C{^1^H} NMR (151 MHz, CD_2_Cl_2_, 303 K): *δ*=141.6, 134.1, 132.3, 131.2, 130.8, 130.6, 129.0, 128.8, 128.3, 127.5, 127.4, 126.5, 125.9, 123.9, 122.2 ppm, IR: $\tilde{\nu }$ =3054, 3018, 2961, 2922, 2872, 2853, 1592, 857, 797, 757 cm^−1^, HRMS (MALDI^+^) *m*/*z*: [*M*+H]^+^: calc. for [C_36_H_22_]^+^: 454.1720, found 454.1659, correct isotope distribution.

**Naphtho[2,3‐c]tetraphene (11)**: According to GP, **10** (200 mg, 609 μmol, 1.00 equiv.) and PtCl_2_ (40.5 mg, 329 μmol, 0.25 equiv.) were dried for 1 h under vacuum. 50 mL of dry toluene (freeze pumped three times) were added and the reaction was stirred at 80 °C for 3 d. The mixture was allowed to cool to room temperature before the organic phase was washed several times with 1 N aqueous HCl. The precipitated solid was filtered and washed with 1 N HCl, EtOAc, H_2_O and ethanol. The crude product was purified by flash chromatography (SiO_2_, PE/EA 10:1) to obtain **11** as a pale yellow solid (160 mg, 487 μmol, 80 %). *R*_f_=0.25 (SiO_2_, petroleum ether, ethyl acetate 10:1, *v*/*v*), M.p.: >300 °C, ^1^H NMR (600 MHz, [D_8_]THF, 323 K): *δ*=9.42 (s, 2 H), 8.94 (d, *J=*9.11 Hz, 2 H), 8.58 (s, 2 H), 8.23–8.21 (m, 4 H), 8.12–8.08 (m, 2 H), 7.57–7.52 ppm (m, 4 H), ^13^C{^1^H} NMR (151 MHz, [D_8_]THF, 323 K): *δ*=133.4, 133.1, 132.1, 130.4, 129.5, 128.8, 128.6, 127.6, 126.6, 126.5, 123.3, 122.4 ppm, IR: $\tilde{\nu }$ =3046, 2961, 1467, 1354, 1260, 1088, 1074, 1053, 1017, 1005, 957, 895, 879, 841, 811 cm^−1^, HRMS (MALDI^+^) *m*/*z*: [*M*+H]^+^: calc. for [C_26_H_16_]^+^: 328.1252, found 328.1249, correct isotope distribution.

**6,6′‐Binaphtho[2,3‐c]tetraphene (13)**: According to GP, **12** (120 mg, 183 μmol, 1.00 equiv.) and PtCl_2_ (14.6 mg, 55.0 μmol, 0.25 equiv.) were dried for 1 h under vacuum. Dry toluene (20 mL, freeze pumped three times) were added and the reaction was stirred at 80 °C for 3 d. The mixture was allowed to cool to room temperature before the organic phase was washed several times with 1 N HCl solution. The precipitated solid was filtered and washed with 1 N HCl, EtOAc, H_2_O and ethanol. The crude product was purified by flash chromatography (SiO_2_, PE/EA 10:1) to obtain **13** as a pale yellow solid (105 mg, 160 μmol, 87 %). *R*_f_=0.24 (SiO_2_, petroleum ether, ethyl acetate 10:1, *v*/*v*), M.p.: >300 °C, ^1^H NMR (600 MHz, [D_8_]THF, 323 K): *δ*=9.66 (s, 1 H), 9.18 (d, *J=*9.35 Hz, 2 H), 8.77 (s, 2 H), 8.51 (s, 2 H), 8.35 (s, 2 H), 8.32 (d, *J=*8.53 Hz, 2 H), 8.27 (s, 2 H), 8.22 (d, *J=*9.38 Hz, 2 H), 8.04 (d, *J=*8.25 Hz, 2 H), 7.79 (d, *J=*8.53 Hz, 2 H), 7.58 (t, *J=*7.25 Hz, 3 H), 7.52 (t, *J=*7.87 Hz, 2 H), 7.19 (t, *J=*7.41 Hz, 2 H), 6.92 (t, *J=*7.95 Hz, 2 H), 6.69 ppm (d, *J=*8.25 Hz, 2 H), ^13^C{^1^H} NMR (151 MHz, [D_8_]THF, 323 K): *δ*=142.5, 133.7, 133.5, 132.9, 132.0, 131.7, 131.5, 130.1, 130.0, 130.0, 129.8, 129.5, 129.0, 128.7, 128.5, 127.7, 127.6, 127.0, 126.8, 126.5, 126.5, 125.7, 123.6, 122.7 ppm, IR: $\tilde{\nu }$ =048, 2957, 1672, 1600, 1587, 1474, 1282, 1258, 1071, 1006, 953, 905, 885, 861, 803 cm^−1^, HRMS (MALDI^+^) *m*/*z*: [*M*+H]^+^: calc. for [C_52_H_30_]^+^: 654.2348, found 654.2448, correct isotope distribution.

## Conflict of interest

The authors declare no conflict of interest.

## Supporting information

As a service to our authors and readers, this journal provides supporting information supplied by the authors. Such materials are peer reviewed and may be re‐organized for online delivery, but are not copy‐edited or typeset. Technical support issues arising from supporting information (other than missing files) should be addressed to the authors.

Supplementary
