# Supplementary material for: Chrysene‐Based Blue Emitters
Source: Chemistry. 2020 Oct 19;26(66):15089–93. doi: 10.1002/chem.202001808 (PMC7756344; doi:10.1002/chem.202001808)
Supplement: Supplementary file 1 — Supplementary [file CHEM-26-15089-s001.pdf]

Chemistry–A European Journal

Supporting Information

Chrysene-Based Blue Emitters

Marvin Nathusius+,[a, b, c] Barbara Ejlli+,[a, b, c] Frank Rominger,[b] Jan Freudenberg,[b, c] Uwe H. F. Bunz,[b] and Klaus Müllen*[a]

Table of contents

S1. General remarks 1

S2. Synthesis 4

S3. Mass spectra 18

S4. UV-Vis spectra 21

S5. Stability measurement 22

S6. Cyclic voltammetry 23

S7. Crystal structure and packing 24

S8. Calculations 25

S9. NMR spectra 26

S10. Crystallographic data 38

S11. References 44

S1. General remarks

Reagents and solvents for synthesis

All reagents and solvents were obtained from commercial suppliers and used without further purification. Deuterated solvents for NMR analysis were purchased from Sigma-Aldrich Laborchemikalien gmbH. Absolute solvents were used directly from a solvent system (MB SPS-800) containing sufficient drying agents.

Column chromatography

Flash column chromatography was carried out using silica gel (grain size 0.04 - 0.063 mm) produced by Machery Nagel. As mobile phase were used the solvents named in the synthetic procedure. For thin layer chromatography Polygram Sil g/UV 254 plates from Macherey Nagel were used and examined under UV-light irradiation (254 nm and 365 nm).

Nuclear magnetic resonance spectroscopy

All NMR spectra were recorded in deuterated solvents (CD_2Cl_2, THF-d_8) at room temperature (if not stated otherwise) on a Bruker Avance III (400 MHz), Bruker Avance III (500 MHz) or Bruker Avance III (600 MHz). ^{13}C NMR spectra were measured proton decoupled if not stated otherwise. Chemical shifts δ are reported in part per million (ppm) and coupling constants J in Hz. All spectra were referenced to solvent signal.[1] For the multiplicities, the following abbreviations are used: s = singlet, d = doublet, t = triplet, m = multiplet. The spectra were processed and integrated using ACD/Spectrus processor.

Mass spectrometry

High-resolution mass spectra (HRMS) were obtained by matrix-assisted laser desorption ionization (MALDI) using DCTB or TCNQ as matrix or direct analysis in real time (DART) experiments on Bruker ApexQe hybrid 9.4 T FT-ICR or Bruker AutoFlex Speed time-of-flight spectrometers.

IR spectra

IR spectra were recorded on a JASCO FT/IR-4100 using the neat compounds at room temperature. The data was processed using JASCO Spectra Manager™ II and all signals are reported in wavelength [cm^{-1}].

X-ray single-crystal structure analysis

X-ray single-crystal structure analyses were measured on a Bruker Smart APEX-II Quazar Area Detector diffractometer. Diffraction intensities were corrected for Lorentz and polarization effects. An empirical absorption correction was applied using SADABS based on the Laue symmetry of reciprocal space. Heavy atom diffractions were solved by direct methods and refined against F^2 with the full matrix least square algorithm. Hydrogen atoms were either isotropically refined or calculated. The structures were solved and refined using the SHELXTL software package.

Quantum yields

Quantum yields were measured using an Ulbricht sphere with a PTI QuantaMaster 40 equipped with an integration sphere (diameter 6″, coated with Spectraflect).

UV-Vis and fluorescence spectra

All UV-Vis spectra were recorded on a JASCO UV-Vis 660 and fluorescence spectra were recorded on a Jasco FP-6500.

Computational studies

Computational studies were carried out using DFT/TD-DFT calculations on Turbomole 6.3.1 and gaussian16. Geometry optimizations were performed using the B3LYP functional and def2-TZVP basis set. At this geometry, the absolute energy were assigned by a single-point approach at the B3LYP/6-311++G** level of theory.[2]

Melting points

Melting points were determined in open glass capillaries with a Melting Point Apparatus MEL-TEMP (Electrothermal, Rochford, UK).

Cyclic voltammetry

The cyclic voltammetry (CV) measurements were carried out using a glassy carbon electrode, a platinum/titanium wire auxiliary electrode and a silver wire reference electrode with a 0.1 mol/L NBu_4PF_6 solution in degassed and dry THF. As the reference redox system ferrocene/ferrocenium was used and the internal standard (−5.1 eV) at room temperature.[3]

S2. Synthesis

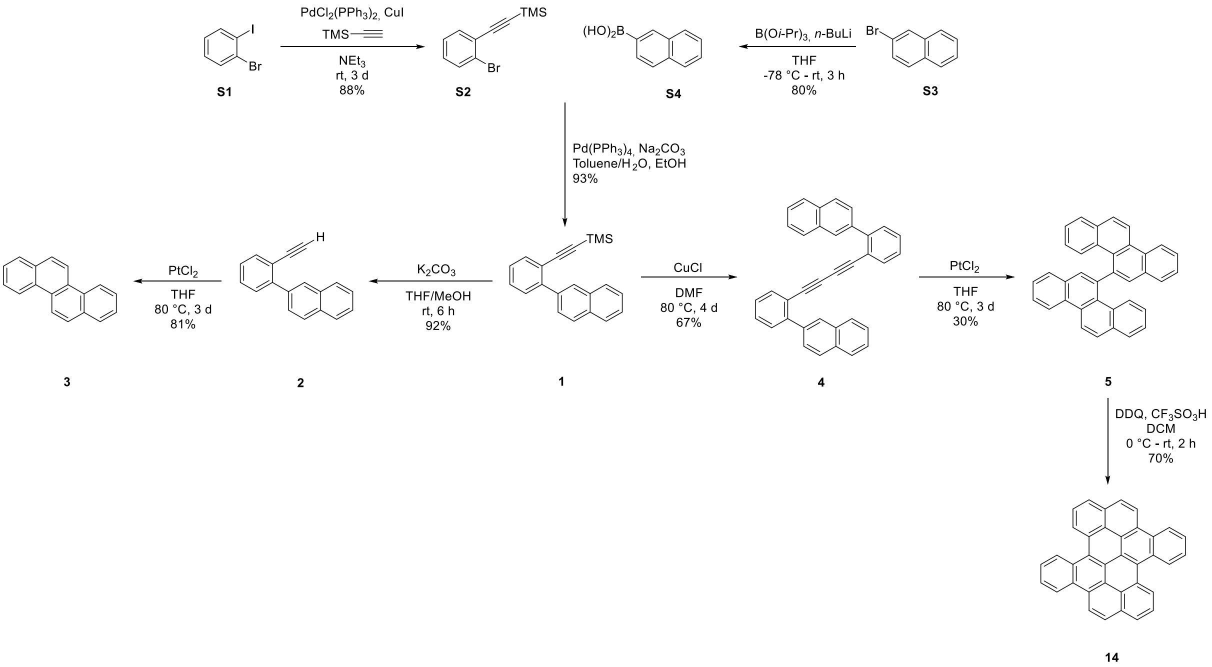

Scheme S1: Synthetic procedure for the synthesis of chrysene (**3**), 5,5’-bichrysenyl (**5**) and **14**.

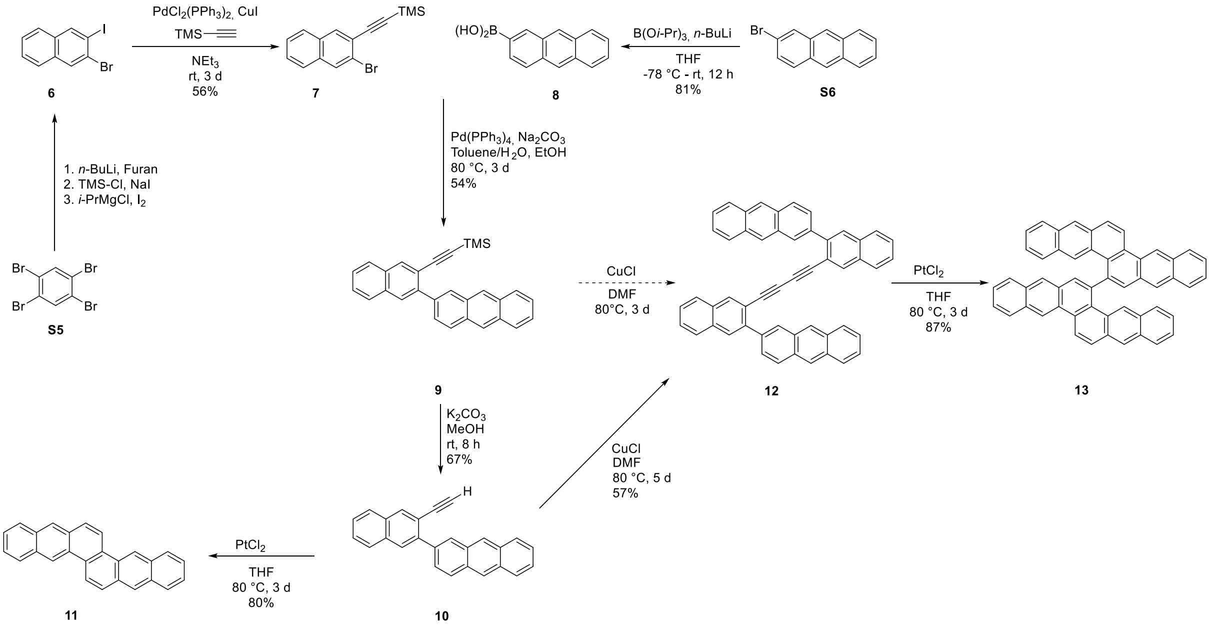

Scheme S2: Synthetic procedure for the synthesis of naphtho[2,3-*c*]tetraphene (**11**) and 6,6'-binaphtho[2,3-*c*]tetraphene (**13**).

Compounds **S4** and **6** were synthesized according to literature procedure.[4],[5]

((2-Bromophenyl)ethynyl)trimethylsilane (S2)

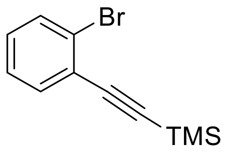

S2

In an oven dried Schlenk flask, 2-bromoiodobenzene **S1** (10.0 g, 35.3 mmol, 1.00 eq.) was added to a solution of 100 mL Et_3N and 10 mL THF. The solution was degassed 1 h by bubbling argon through the solution before CuI (269 mg, 1.41 mmol, 0.04 eq.), $PdCl_2(PPh_3)_2$ (651 mg, 2.12 mmol, 0.06 eq.) and TMSA (4.17 g, 42.4 mmol, 1.20 eq.) were added. The mixture was stirred at rt for 3 d and quenched with 10 mL of NH_4Cl. The mixture was extracted three times with EtOAc and the combined organic phases were dried over Na_2SO_4. The solvent was removed under reduced pressure and the residue was purified by flash chromatography (SiO_2/PE) to obtain the product **S2** as a yellow oil (7.92 g, 31.3 mmol, 88%).

^{1}H NMR (500 MHz, CD_2Cl_2, 295 K): δ [ppm] = 7.59 (d, J=7.78 Hz, 1H), 7.49 (d, J=7.55 Hz, 1H), 7.27 (t, J=7.32 Hz, 1H), 7.19 (t, J=7.55 Hz, 1H), 0.27 (s, 9H).

^{13}C{^{1}H} NMR (126 MHz, CD_2Cl_2, 295 K): δ [ppm] = 134.1, 132.9, 130.2, 127.6, 126.0, 125.7, 103.4, 100.1, 0.0.

All analytical data is in good agreement with the literature.[6]

Trimethyl((2-(naphthalen-2-yl)phenyl)ethynyl)silane (1)

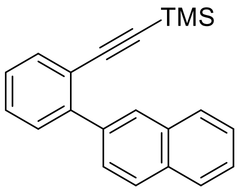

1

In an oven dried Schlenk flask, 2-naphtaleneboronic acid **S4** (2.58 g, 15.0 mmol, 1.00 eq.) and Na_2CO_3 (9.54 g, 90.0 mmol, 6.00 eq.) were dried for 10 minutes under vacuum. **S2** (3.80 g, 15.0 mmol, 1.00 eq.) and 300 mL toluene, 75 mL EtOH and H_2O were added and the mixture was degassed 30 min by bubbling argon through the solution. $Pd(PPh_3)_4$ (1.73 g, 1.50 mmol, 0.10 eq.) was added and the mixture was stirred at 80 °C for 3 d, allowed to cool to room temperature and extracted three times with EtOAc. The combined organic layers were washed with brine, dried over Na_2SO_4 and the solvent was removed under reduced pressure. The crude product was purified by flash chromatography (SiO_2/PE) to obtain the product **1** as a yellow oil (4.20 g, 14.0 mmol, 93%).

^{1}H NMR (500 MHz, CD_2Cl_2, 295 K): δ [ppm] = 8.11 (s, 1 H), 7.90 (m, 3H), 7.79 (d, J=8.32 Hz, 1H), 7.62 (d, J=7.64 Hz, 1H), 7.52 (m, 3 H), 7.45 (t, J=7.30 Hz, 1 H), 7.34 (t, J=7.47 Hz, 1H), 0.10 (s, 9H).

^{13}C{^{1}H} NMR (126 MHz, CD_2Cl_2, 295 K): δ [ppm] = 144.4, 138.2, 134.0, 133.6, 133.2, 130.3, 129.5, 128.7, 128.1, 127.4, 126.6, 122.1, 105.1, 98.2, -0.1.

All analytical data is in good agreement with the literature.[7]

2-(2-Ethynylphenyl)naphthalene (2)

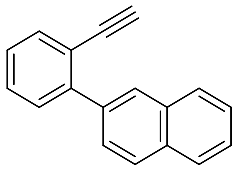

2

In an oven dried Schlenk flask, **1** (400 mg, 1.32 mmol, 1.00 eq.) was dissolved in 10 mL THF/MeOH (1:1) and K_2CO_3 (292 mg, 2.12 mmol, 1.60 eq.) was added. The mixture was stirred at room temperature while the progress of the reaction was monitored by TLC. The reaction was complete after 6 h and the solid was filtered off. The residue was washed several times with DCM, the solvent was removed under reduced pressure and the residue was purified by column chromatography (SiO_2, PE/EA 10:1). The product **2** was obtained as a pale yellow oil (280 mg, 1.22 mmol, 92%).

^{1}H NMR (400 MHz, CD_2Cl_2, 295 K): δ [ppm] = 8.14 (s, 1 H), 8.04 – 7.92 (m, 3 H) 7.83 (dd, J=8.44, 1.65 Hz, 1 H), 7.78 - 7.74 (m, 1 H) 7.62 - 7.54 (m, 3 H) 7.54 - 7.50 (m, 1 H) 7.45 - 7.39 (m, 1 H), 3.18 (s, 1 H).

^{13}C{^{1}H} NMR (101 MHz, CD_2Cl_2, 295 K): δ [ppm] = 145.2, 138.7, 134.8, 134.1, 133.6, 130.7. 129.0, 128.5, 128.4, 128.3, 127.1, 121.5, 84.0, 81.2.

1,4-Bis(2-(naphthalen-2-yl)phenyl)buta-1,3-diyne (4)

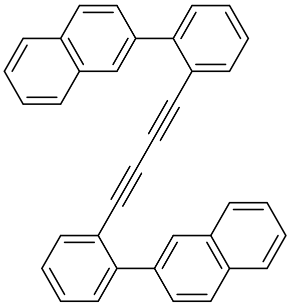

4

1 (1.00 g, 3.33 mmol, 1.00 eq.) was dissolved in 25 mL DMF and CuCl (3.29 g, 33.3 mmol, 10.0 eq.) was added. The reaction was stirred at 80 °C for 4 d and the progress of the reaction was monitored by TLC. The mixture was diluted with 100 mL EtOAc, quenched with 10 mL 1 N HCl and the aqueous phase was extracted three times with EtOAc. The combined organic layers were washed several times with brine, dried over Na_2SO_4 and the solvent was removed under reduced pressure. The crude product was purified by flash chromatography (SiO_2, PE/EA 10:1) to obtain **4** as a colorless solid (505 mg, 1.11 mmol, 67%).

R_f = 0.64 (SiO_2, petroleum ether, ethyl acetate 10:1, *v*/*v*), M.p.: 143 °C

^{1}H NMR (600 MHz, CD_2Cl_2, 295 K): δ [ppm] = 8.03 - 7.98 (m, 2H), 7.91 - 7.79 (m, 6H), 7.74 - 7.67 (m, 2H), 7.66 - 7.60 (m, 2H), 7.57 - 7.45 (m, 8H), 7.38 - 7.31 (m, 2H).

^{13}C{^{1}H} NMR (151 MHz, CD_2Cl_2, 295 K): δ [ppm] = 145.1, 137.6, 134.4, 129.7, 128.4, 128.2, 127.8, 127.6, 127.4, 126.5, 126.4, 120.4, 81.7, 76.7.

HRMS (DART$^+$) m/z: $[M+H]^+$: calc. for $[C_{36}H_{23}]^+$: 455.1794, found 455.1793.

IR: $\tilde{\nu}$ [cm^{-1}] = 3050, 3019, 1601, 1587, 1480, 1464, 1440, 897, 870, 766, 754.

5,5'-Bichrysenyl (5)

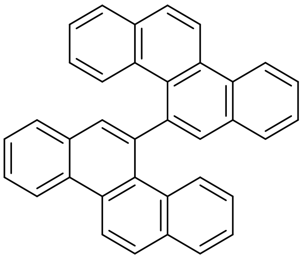

5

In a flame dried Schlenk flask, **4** (600 mg, 1.32 mmol, 1.00 eq.) and $PtCl_2$ (87.7 mg, 329 µmol, 0.25 eq.) were dried for 1 h under vacuum. 20 mL dry toluene (freeze pumped three times) were added and the reaction was stirred at 80 °C for 3 d. The mixture was allowed to cool to room temperature before the solvent was removed under reduced pressure. The crude product was purified by flash chromatography (SiO_2, PE/EA 10:1) to obtain **5** as a pale yellow solid (180 mg, 1.32 mmol, 30%).

R_f = 0.29 (SiO_2, petroleum ether, ethyl acetate 10:1, *v*/*v*), M.p.: > 300 °C

^{1}H NMR (600 MHz, CD_2Cl_2, 295 K): δ [ppm] = 8.98 (d, *J*=9.16 Hz, 2H), 8.94 (d, *J*=8.88 Hz, 2H), 8.28 (d, *J*=9.09 Hz, 2H), 8.16 (d, *J*=9.13 Hz, 2H), 7.98 (d, *J*=8.01 Hz, 2H), 7.80 - 7.74 (m, 4H), 7.66 (s, 2H), 7.62 (td, *J*=7.43, 0.92 Hz, 2H), 7.37 (td, *J*=7.40, 0.99 Hz, 2H), 6.88 (td, *J*=7.85, 1.51 Hz, 2H).

^{13}C{^{1}H} NMR (151 MHz, CD_2Cl_2, 295 K): δ [ppm] = 141.6, 134.1, 132.3, 131.2, 130.8, 130.6, 129.0, 128.8, 128.3, 127.5, 127.4, 126.5, 125.9, 123.9, 122.2.

HRMS (MALDI$^+$) m/z: [M+H]$^+$: calc. for $[C_{36}H_{22}]^+$: 454.1720, found 454.1659.

IR: $\bar{\nu}$ [cm^{-1}] = 3054, 3018, 2961, 2922, 2872, 2853, 1592, 857, 797, 757.

Chrysene (3)

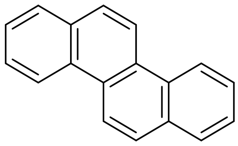

3

In a flame dried Schlenk flask, **2** (300 mg, 1.31 mmol, 1.00 eq.) and $PtCl_2$ (87.4 mg, 329 µmol, 0.25 eq.) were dried for 1 h under vacuum. 20 mL dry toluene (freeze pumped three times) were added and the reaction was stirred at 80 °C for 3 d. The mixture was allowed to cool to room temperature before the solvent was removed under reduced pressure. The crude product was purified by flash chromatography (SiO_2, PE/EA 10:1) to obtain a pale yellow solid **3** (245 mg, 1.06 mmol, 81%).

^{1}H NMR (400 MHz, CD_2Cl_2, 295 K) δ [ppm] = 7.69 - 7.63 (m, 2 H) 7.76 - 7.71 (m, 2 H) 8.07 - 8.00 (m, 4 H) 8.78 - 8.73 (m, 2 H) 8.81 (d, *J*=8.25 Hz, 2 H)

^{13}C{^{1}H} NMR (101 MHz, CD_2Cl_2, 295 K): δ [ppm] = 132.9, 131.2, 129.2, 128.8, 128.0, 127.4, 127.1, 123.8, 121.8.

All analytical data is in good agreement with the literature[7]

Benzo[*a*]dinaphtho[2,1,8-*cde*:1',2',3',4'-*ghi*]perylene (14)

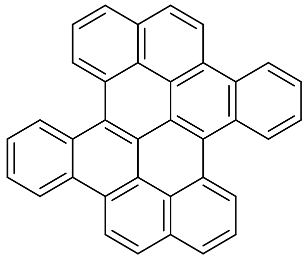

14

In a flame dried Schlenk flask, **5** (36.0 mg, 79.2 µmol, 1.00 eq.) and DDQ (71.9 mg, 316 µmol, 4.00 eq.) were dissolved in 20 mL dry DCM and cooled to -78 °C. To this solution CF_3SO_3H (71.3 mg, 475 µmol, 6.00 eq.) was added dropwise and the reaction mixture was allowed to warm up to room temperature while stirred for additional 2 h. The reaction was quenched with K_2CO_3 and the solvent removed under reduced pressure. The remaining solid was filtered off and washed with H_2O and methanol several times. The product **14** was obtained as a dark red solid (25.0 mg, 55.5 µmol, 70%).

M.p.: > 300 °C

NMR: Due to low solubility, no NMR spectra could be obtained.

HRMS (MALDI$^+$) m/z: $[M+H]^+$: calc. for $[C_{36}H_{18}]^+$: 450.1408, found 450.1378.

IR: $\bar{\nu}$ [cm^{-1}] = 3050, 3019, 1601, 1587, 960, 943, 897, 870, 853, 813.

((3-Bromonaphthalen-2-yl)ethynyl)trimethylsilane (7)

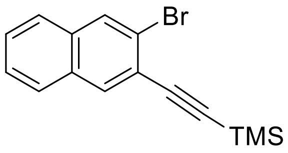

7

In an oven dried Schlenk flask, **6** (6.35 g, 22.2 mmol, 1.00 eq.) was dissolved in 60 mL Et_3N/ 50 mL THF and degassed for 1 h with argon. CuI (169 mg, 888 µmol, 0.04 eq.), $PdCl_2(PPh_3)_2$ (409 mg, 1.33 mmol, 0.06 eq.) and TMSA (2.62 g, 26.6 mmol, 1.20 eq.) were added and the mixture was stirred at rt for 3 d. The reaction was quenched with 10 mL of an aqueous NH_4Cl solution, extracted three times with EtOAc and dried over Na_2SO_4. The solvent was removed under reduced pressure and the crude product purified by flash chromatography (SiO_2, PE) to obtain **7** as a yellow oil (3.78 g, 12.5 mmol, 56%).

^1H NMR (500 MHz, CD_2Cl_2, 295 K): δ [ppm] = 8.10 (s, 1H), 8.04 (s, 1H), 7.79 -7.74 (m, 2H), 7.53 – 7.51 (m, 2H), 0.30 (s, 9H).

$^{13}C\{^1H\}$ NMR (126 MHz, CD_2Cl_2, 295 K): δ [ppm] = 134.3, 134.1, 132.2, 131.5, 128.3, 128.1, 127.5, 127.3, 122.9, 122.1, 103.7, 99.8, 0.0.

All analytical data is in good agreement with the literature.[8]

((3-(Anthracen-2-yl)naphthalen-2-yl)ethynyl)trimethylsilane (9)

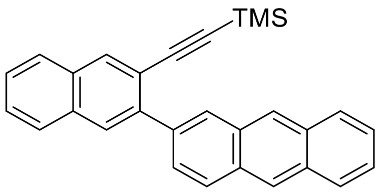

9

In an oven dried Schlenk flask, 2-anthraceneboronic acid **8** (1.03 g, 4.62 mmol, 1.00 eq.) and Na_2CO_3 (2.94 g, 27.7 mmol, 6.00 eq.) were dried for 10 minutes under vacuum. **7** (1.40 g, 4.62 mmol, 1.00 eq.) and 240 mL toluene, 60 mL EtOH and H_2O were added and the mixture was degassed 30 min with argon. $Pd(PPh_3)_4$ (533 mg, 461 µmol, 0.10 eq.) was added and the mixture was stirred at 80 °C for 3 d, allowed to cool to room temperature and extracted three times with EtOAc. The combined organic layers were washed with brine, dried over Na_2SO_4 and the solvent was removed under reduced pressure. The crude product was purified by flash chromatography (SiO_2/PE) to obtain the product **9** as a pale yellow solid (1.01 g, 2.50 mmol, 54%).

R_f = 0.73 (SiO_2, petroleum ether), M.p.: 147 °C

^{1}H NMR (400 MHz, CD_2Cl_2, 295 K): δ [ppm] = 8.51 (d, J=3.12 Hz, 2H), 8.34 (s, 1H), 8.19 (s, 1H), 8.12 - 8.02 (m, 4H), 7.92 - 7.87 (m, 3H), 7.57 - 7.53 (m, 2H), 7.49-7.46 (m, 2H), 0.11 (s, 9H).

^{13}C{^{1}H} NMR (101 MHz,CD_2Cl_2, 295 K): δ [ppm] = 141.0, 137.9, 134.2, 133.8, 132.7, 132.6, 132.5, 132.1, 131.6, 129.2, 128.7, 127.9, 128.4, 127.2, 126.1, 126.0, 120.5, 105.4, 99.0, 0.1.

HRMS (DART$^+$) m/z: [M+H]$^+$: calc. for [$C_{29}H_{25}Si$]$^+$: 401.1720, found 401.1718.

IR: $\tilde{\nu}$ [cm^{-1}] = 3046, 3015, 2957, 2898, 2152, 1626, 1588, 1246, 905, 896, 887.

2-(3-Ethynylnaphthalen-2-yl)anthracene (10)

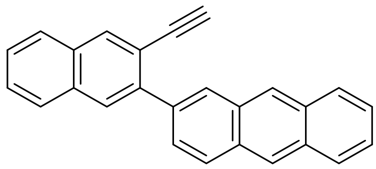

10

In an oven dried Schlenk flask, **9** (2.75 g, 6.86 mmol, 1.00 eq.) was dissolved in 140 mL THF/MeOH (1:1) and K_2CO_3 (1.52 g, 10.9 mmol, 1.60 eq.) was added. The mixture was stirred at rt while the progress of the reaction was monitored by TLC. The reaction was complete after 8 h and the solid was filtered off. The crude product was extracted with DCM, the solvent was removed under reduced pressure and the residue was purified by column chromatography (SiO_2, PE/EA 10:1). The product **10** was obtained as a colorless solid (1.48 g, 4.57 mmol, 67%).

R_f = 0.65 (SiO_2, petroleum ether, ethyl acetate 10:1, *v*/*v*), M.p.: 126 °C

^{1}H NMR (500 MHz, CD_2Cl_2, 295 K): δ [ppm] = 8.52 (d, *J*=10.08 Hz, 2H), 8.30 (s, 1H), 8.24 (s, 1H), 8.13 - 8.09 (m, 1H), 8.08 - 8.04 (m, 2H), 8.01 (s, 1H), 7.94 -7.88 (m, 2H), 7.86 - 7.82 (m, 1H), 7.60 - 7.54 (m, 2H), 7.51 (m, 1H), 7.52 - 7.48 (m, 1H), 3.20 (s, 1H).

^{13}C{^{1}H} NMR (126 MHz, CD_2Cl_2, 295 K): δ [ppm] = 140.6, 137.3, 134.4, 133.4, 132.1, 131.5, 131.0, 128.9, 128.3, 128.1, 127.6, 127.2, 126.1, 125.6, 119.0, 83.4, 80.8, 54.0.

HRMS (DART$^+$) m/z: $[M+H]^+$: calc. for $[C_{26}H_{17}]^+$: 329.1325, found 329.1324.

IR: $\bar{\nu}$ [cm^{-1}] = 3287, 3048, 3019, 1624, 1589, 952, 933, 910, 891, 887, 868, 850.

1,4-Bis(3-(anthracen-2-yl)naphthalen-2-yl)buta-1,3-diyne (12)

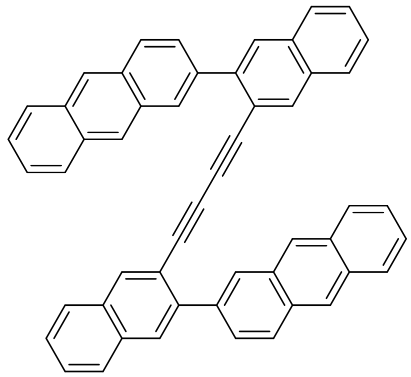

12

10 (140 mg, 426 µmol, 1.00 eq.) was dissolved in 25 mL DMF and CuCl (422 mg, 4.26 mmol, 10.0 eq.) was added. The reaction was stirred at 80 °C for 5 d and the progress of the reaction was monitored by TLC. The mixture was diluted with 100 mL EtOAc, quenched with 10 mL 1N HCl and the aqueous phase was extracted three times with EtOAc. The precipitated solid was filtered and washed several times with EtOAc, H_2O and ethanol. The crude product was purified by flash chromatography (SiO_2, PE/EA 10:1) to obtain **12** as a yellow solid (81 mg, 122 µmol, 57%).

R_f = 0.51 (SiO_2, petroleum ether, ethyl acetate 10:1, *v*/*v*), M.p.: 248 °C

^{1}H NMR (600 MHz, THF-d_8, 328 K): δ [ppm] = 8.51 (s, 2H), 8.34 (s, 2H), 8.28 - 8.24 (m, 2H), 8.21 (s, 2H), 8.05 – 7.97 (m, 6H), 7.94 (d, *J* = 8.80 Hz, 2H), 7.91 - 7.88 (m, 2H), 7.83 (d, *J* = 7.98 Hz, 2H), 7.81 - 7.75 (m, 2H), 7.54 - 7.48 (m, 4H), 7.47 - 7.42 (m, 4H).

^{13}C{^{1}H} NMR (151 MHz, THF-d_8, 328 K): δ [ppm] = 141.9, 138.2, 135.9, 134.7, 133.3, 132.8, 132.2, 129.7, 129.2, 129.1, 128.5, 128.3, 127.6, 127.5, 126.8, 126.2, 119.7, 83.0, 78.4.

HRMS (DART$^+$) m/z: [M+H]$^+$: calc. for $[C_{52}H_{31}]^+$: 655.2420, found 655.2418.

IR: $\tilde{\nu}$ [cm^{-1}] = 3050, 3016, 1668, 1488, 1322, 1293, 929, 881, 740, 707.

Naphtho[2,3-*c*]tetraphene (11)

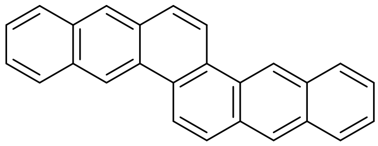

11

In a flame dried Schlenk flask, **10** (200 mg, 609 µmol, 1.00 eq.) and $PtCl_2$ (40.5 mg, 329 µmol, 0.25 eq.) were dried for 1 h under vacuum. 50 mL dry toluene (freeze pumped three times) were added and the reaction was stirred at 80 °C for 3 d. The mixture was allowed to cool to room temperature before the organic phase was washed several times with 1N HCl solution. The precipitated solid was filtered and washed with 1N HCl, EtOAc, H_2O and ethanol. The crude product was purified by flash chromatography (SiO2, PE/EA 10:1) to obtain **11** as a pale yellow solid (160 mg, 487 µmol, 80%).

R_f = 0.25 (SiO_2, petroleum ether, ethyl acetate 10:1, *v*/*v*), M.p.: > 300 °C

^{1}H NMR (600 MHz, THF-d_8, 323 K): δ [ppm] = 9.42 (s, 2H), 8.94 (d, *J*=9.11 Hz, 2H), 8.58 (s, 2H), 8.23 - 8.21 (m, 4H), 8.12 - 8.08 (m, 2H), 7.57 - 7.52 (m, 4H).

^{13}C{^{1}H} NMR (151 MHz, THF-d_8, 323 K): δ [ppm] = 133.4, 133.1, 132.1, 130.4, 129.5, 128.8, 128.6, 127.6, 126.6, 126.5, 123.3, 122.4.

HRMS (MALDI$^+$) m/z: [M+H]$^+$: calc. for [$C_{26}H_{16}$]$^+$: 328.1252, found 328.1249.

IR: $\bar{\nu}$ [cm^{-1}] = 3046, 2961, 1467, 1354, 1260, 1088, 1074, 1053, 1017, 1005, 957, 895, 879, 841, 811.

6,6'-Binaphtho[2,3-*c*]tetraphene (13)

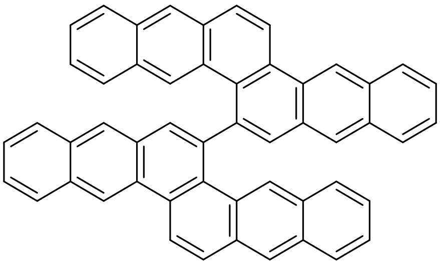

13

In a flame dried Schlenk flask, **12** (120 mg, 183 µmol, 1.00 eq.) and $PtCl_2$ (14.6 mg, 55.0 µmol, 0.25 eq.) were dried for 1 h under vacuum. 20 mL dry toluene (freeze pumped three times) were added and the reaction was stirred at 80 °C for 3 d. The mixture was allowed to cool to room temperature before the organic phase was washed several times with 1N HCl solution. The precipitated solid was filtered and washed with 1N HCl, EtOAc, H_2O and ethanol. The product **13** was obtained as a pale yellow solid (105 mg, 160 µmol, 87%).

R_f = 0.24 (SiO_2, petroleum ether, ethyl acetate 10:1, *v*/*v*), M.p.: > 300 °C

^{1}H NMR (600 MHz, THF-d_8, 323 K): *δ* [ppm] = 9.66 (s, 1H), 9.18 (d, *J*=9.35 Hz, 2H), 8.77 (s, 2H), 8.51 (s, 2H), 8.35 (s, 2H), 8.32 (d, *J*=8.53 Hz, 2H), 8.27 (s, 2H), 8.22 (d, *J*=9.38 Hz, 2H), 8.04 (d, *J*=8.25 Hz, 2H), 7.79 (d, *J*=8.53 Hz, 2H), 7.58 (t, *J*=7.25 Hz, 3H), 7.52 (t, *J*=7.87 Hz, 2H), 7.19 (t, *J*=7.41 Hz, 2H), 6.92 (t, *J*=7.95 Hz, 2H), 6.69 (d, *J*=8.25 Hz, 2H).

^{13}C{^{1}H} NMR (151 MHz, THF-d_8, 323 K): *δ* [ppm] = δ 142.5, 133.7, 133.5, 132.9, 132.0, 131.7, 131.5, 130.1, 130.0, 129.8, 129.5, 129.0, 128.7, 128.5, 127.7, 127.6, 127.0, 126.8, 126.5, 125.7, 123.6, 122.7.

HRMS (MALDI$^+$) m/z: [M+H]$^+$: calc. for $[C_{52}H_{30}]^+$: 654.2348, found 654.2448.

IR: $\tilde{\nu}$ [cm^{-1}] = 3048, 2957, 1672, 1600, 1587, 1474, 1282, 1258, 1071, 1006, 953, 905, 885, 861, 803.

S3. Mass spectra

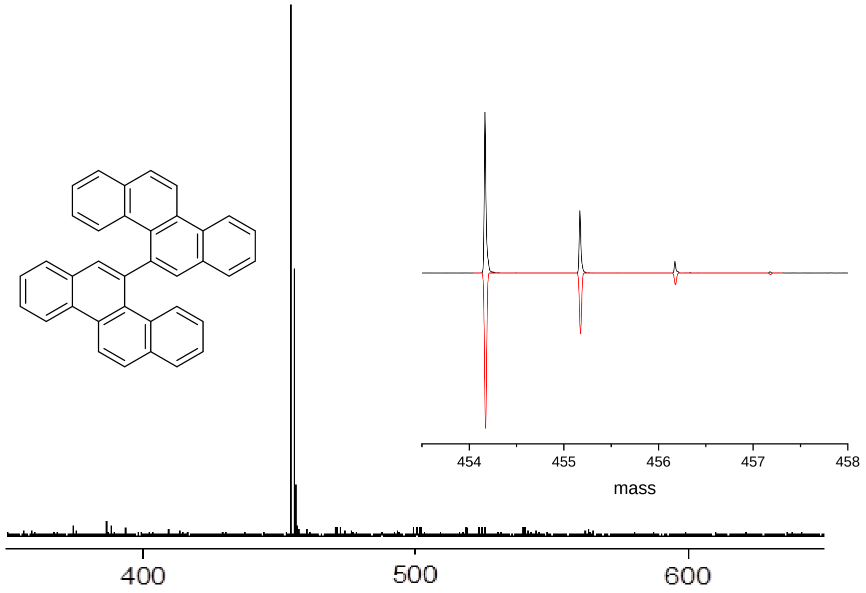

Figure S1: HR-MALDI of 5,5´-bichrysenyl (**5**).

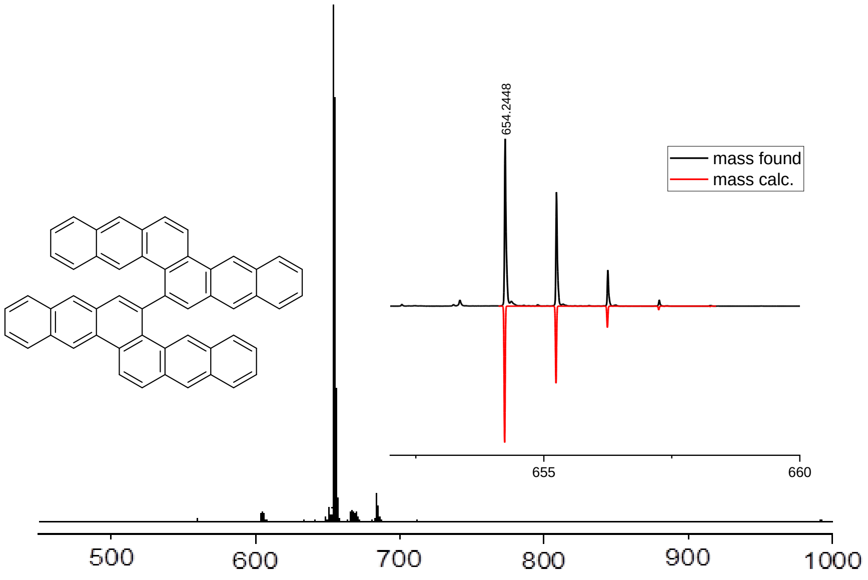

Figure S2: HR-MALDI of 6,6'-binaphtho[2,3-*c*]tetraphene (**13**).

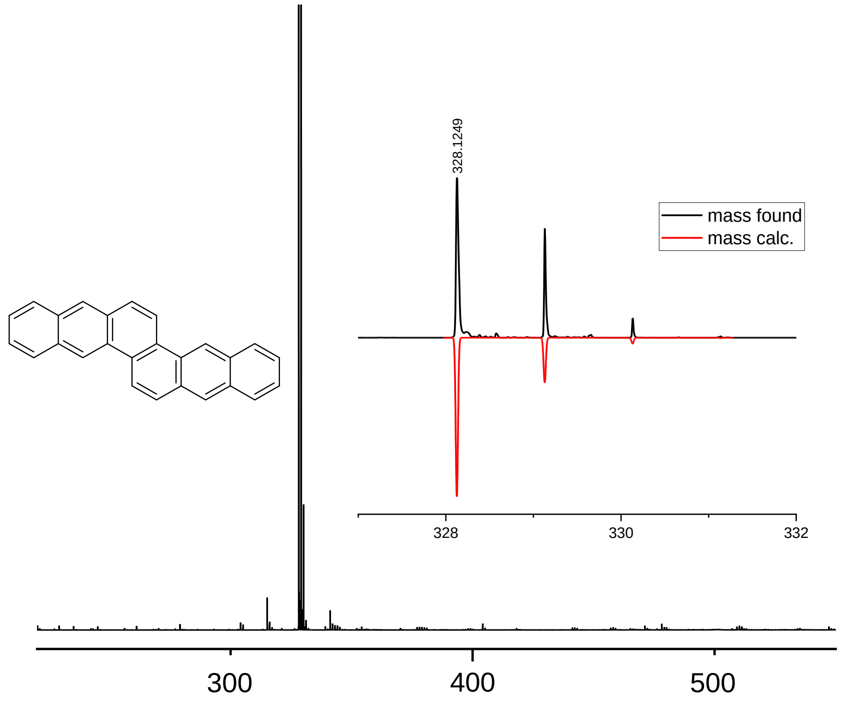

Figure S3: HR-MALDI of binaphtho[2,3-*c*]tetraphene (**11**).

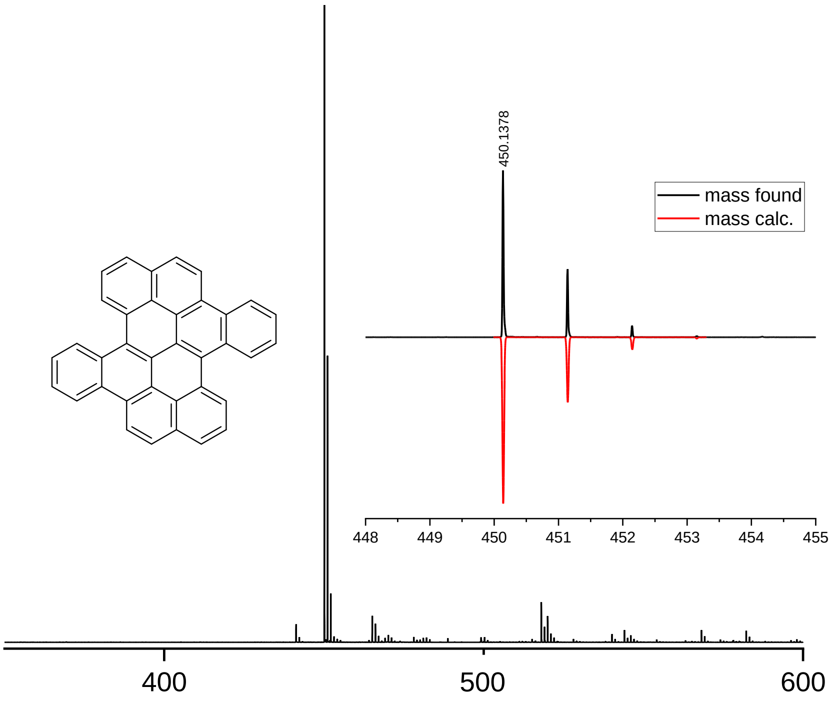

Figure S4: HR-MALDI of **14**.

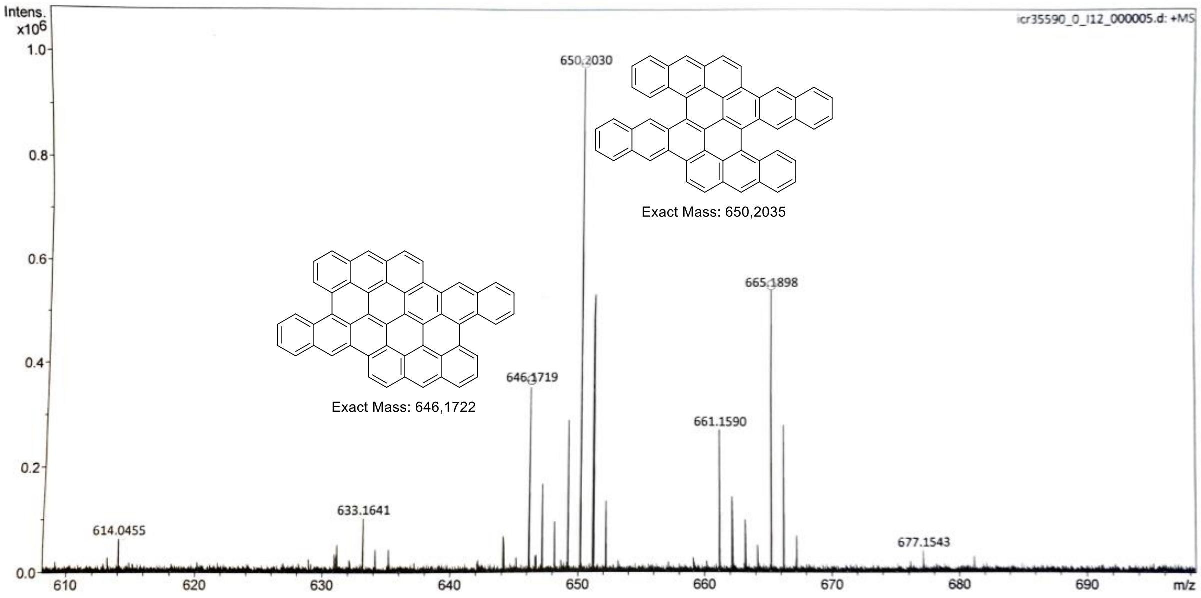

Figure S5: Mass spectra for the cyclodehydrogenation leading to **15**.

S4. UV-Vis spectra

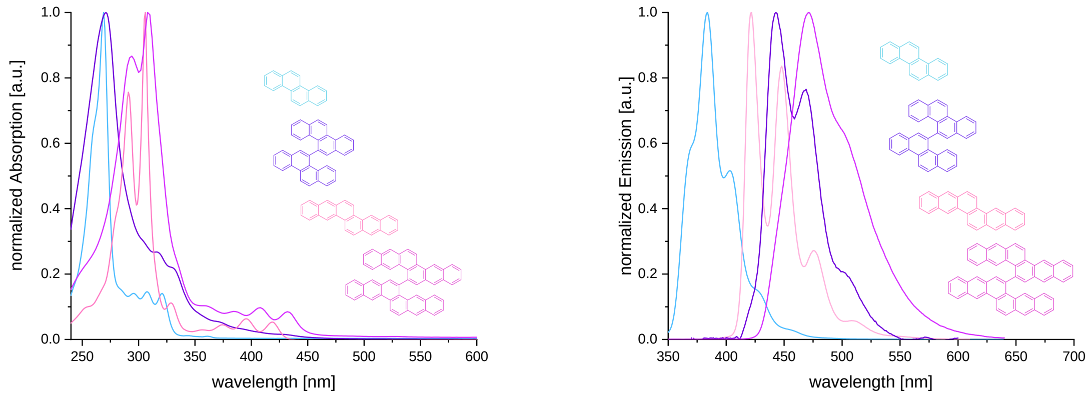

Figure S6: Absorption (left) and emission (right) spectra in *n*-hexane.

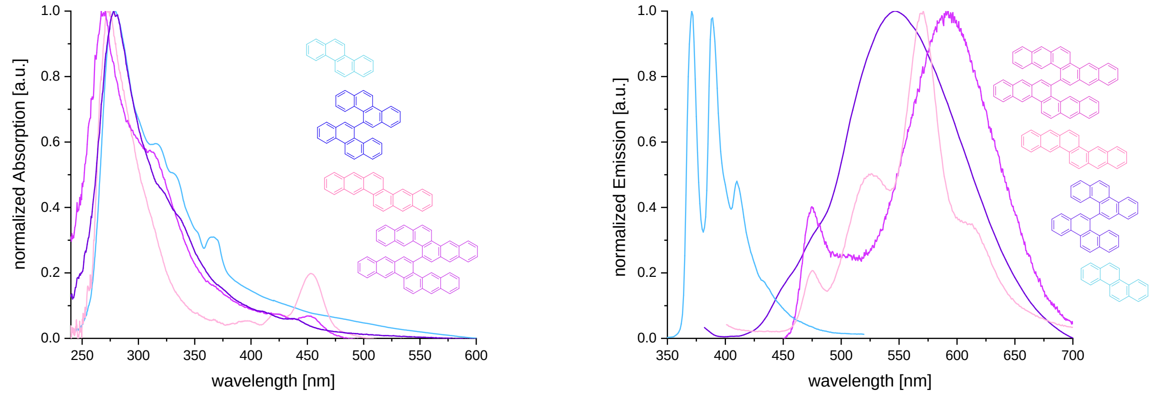

Figure S7: Absorption (left) and emission (right) spectra on thin films.

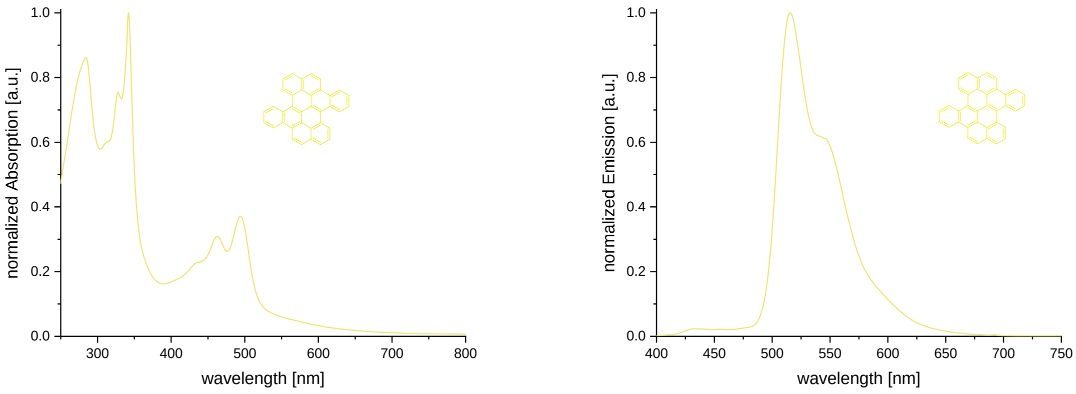

Figure S8: Absorption (left) and emission (right) spectra of **14** in *n*-hexane.

S5. Stability measurement

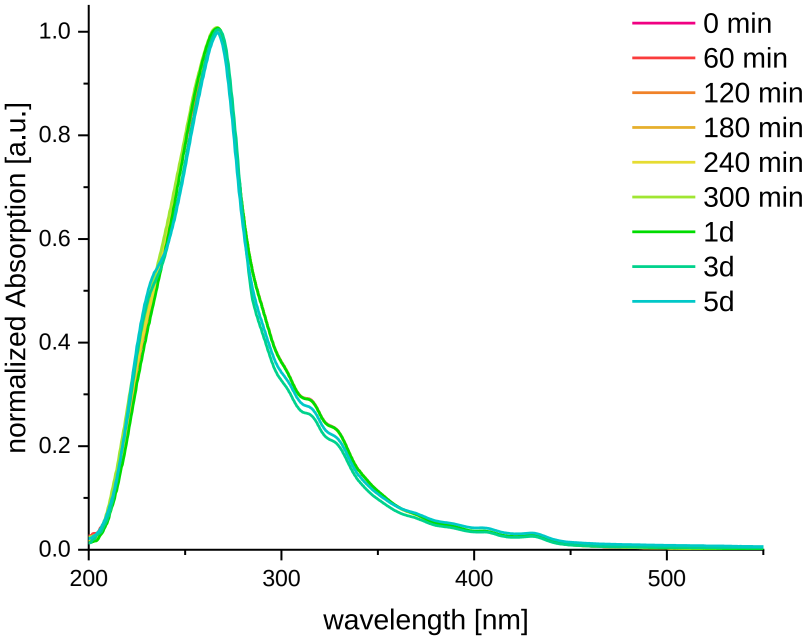

Figure S9: Absorption spectra of **5** under irradiation with 365 nm and oxygen atmosphere in *n*-hexane.

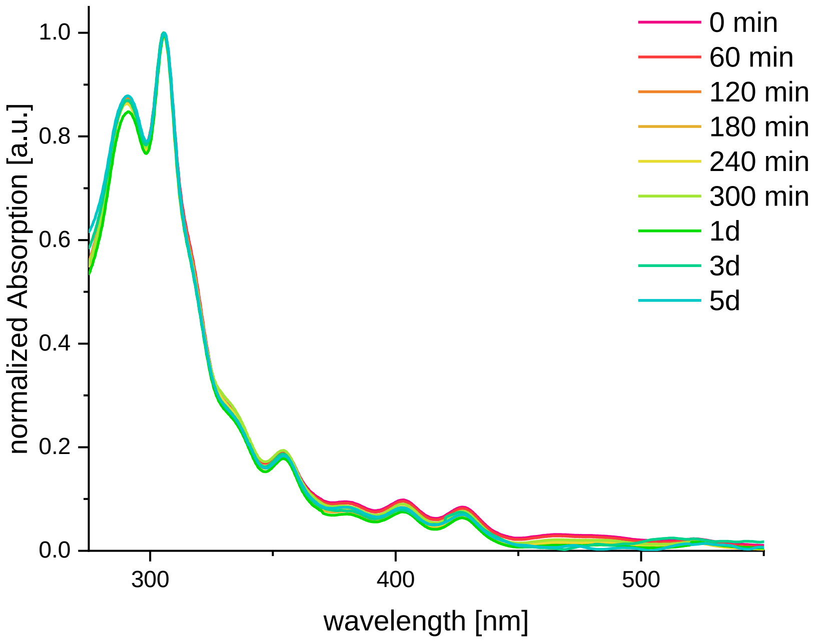

Figure S10: Absorption spectra of **13** under irradiation with 365 nm and oxygen atmosphere in *n*-hexane.

S6. Cyclic voltammetry

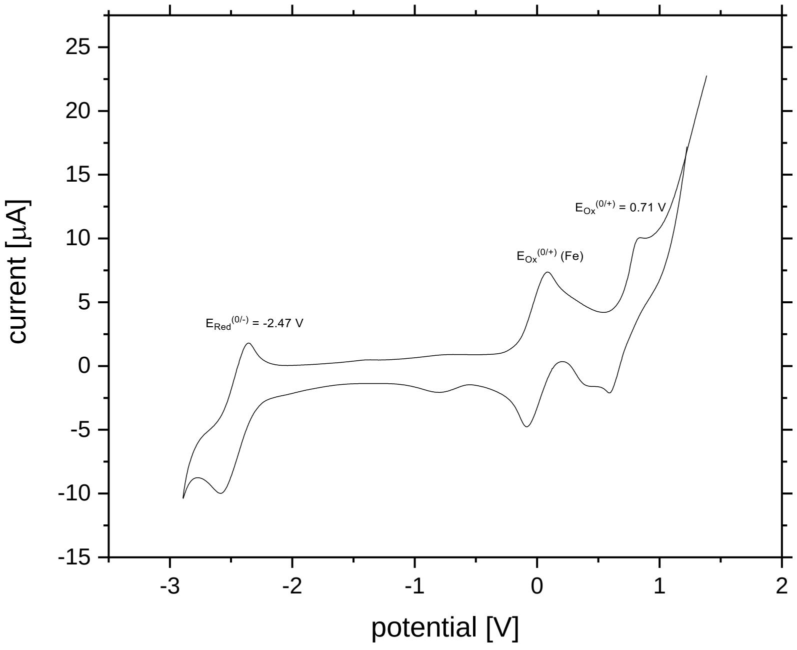

Figure S11: Cyclovoltammogram of naphtho[2,3-*c*]tetraphene **11**.

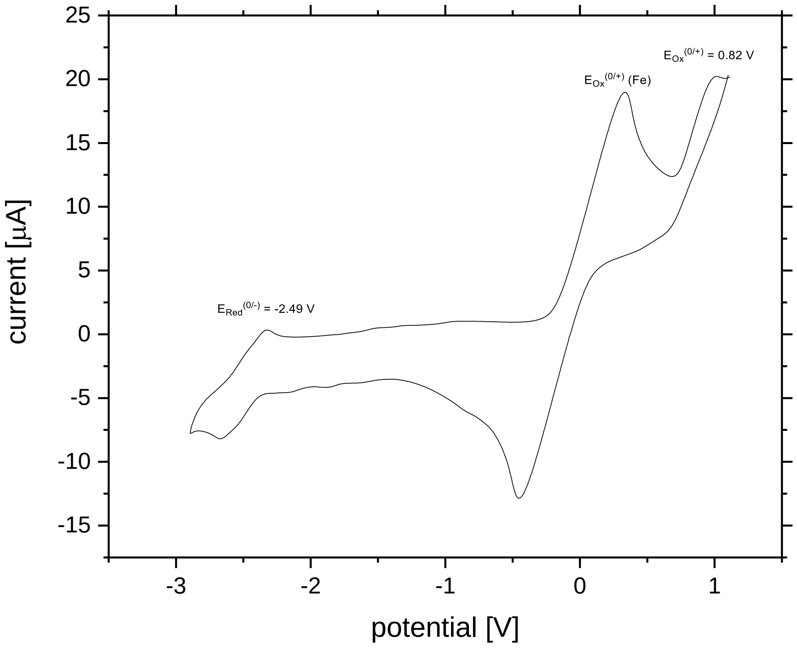

Figure S12: Cyclovoltammogram of 6,6'-binaphtho[2,3-*c*]tetraphene **13**.

Table S1: Experimental and calculated HOMO-LUMO values and gaps for **3**, **5**, **11** and **13**.

	IP_{CV}[a]/ HOMO calc.[c] [eV]	EA_{CV}[b]/ LUMO calc.[c] [eV]	S0/S1 calc.[d] [eV]	λ_{onset}[e]/gap calc. [eV]
**3**	--[f]/-5.71	--[f]/-1.81	3.75	3.75/3.90
**5**	--[f]/-5.56	--[f]/-1.83	3.54	3.56/3.73
**11**	-5.52/-5.19	-2.63/-2.44	2.76	2.89/2.75
**13**	-5.37/-5.13	-2.60/-2.46	2.52	2.77/2.67

[a] $IP_{CV} = EA_{CV} - \lambda_{onset}$. [b] $EA_{CV} = -e \times (5.1\ V + E_{red})$[3], E_{red} was obtained from CV. [c] Frontier molecular orbital energies were obtained from quantum-chemical calculations with Gaussian16 B3LYP/def2SVP//Gaussian16 B3LYP/def2TZVP. [d] TD-DFT calculations were performed by using the optimized geometries in the ground state. [e] λ_{onset} in *n*-hexane. [f] No redox events were observed both for chrysene (**3**) and its biaryl **5** under our conditions.

S7. Crystal structure and packing

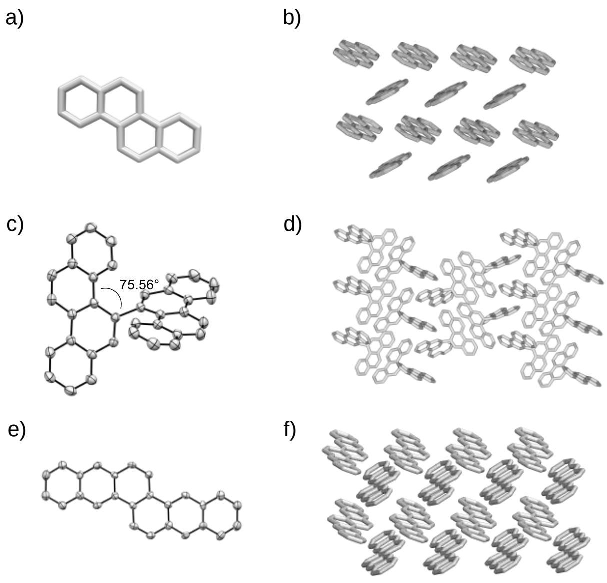

Figure S13: Crystal structure and packing of chrysene (**3**) (a,b)[9], 5,5'-bichrysenyl (**5**) (c,d) and naphtho[2,3-*c*]tetraphene (**11**) (e,f). **3** and **11** both pack in a herringbone motiv, while 5,5'-bichrysenyl (**5**) adopts a torsional angle of ~76°.

S8. Calculations

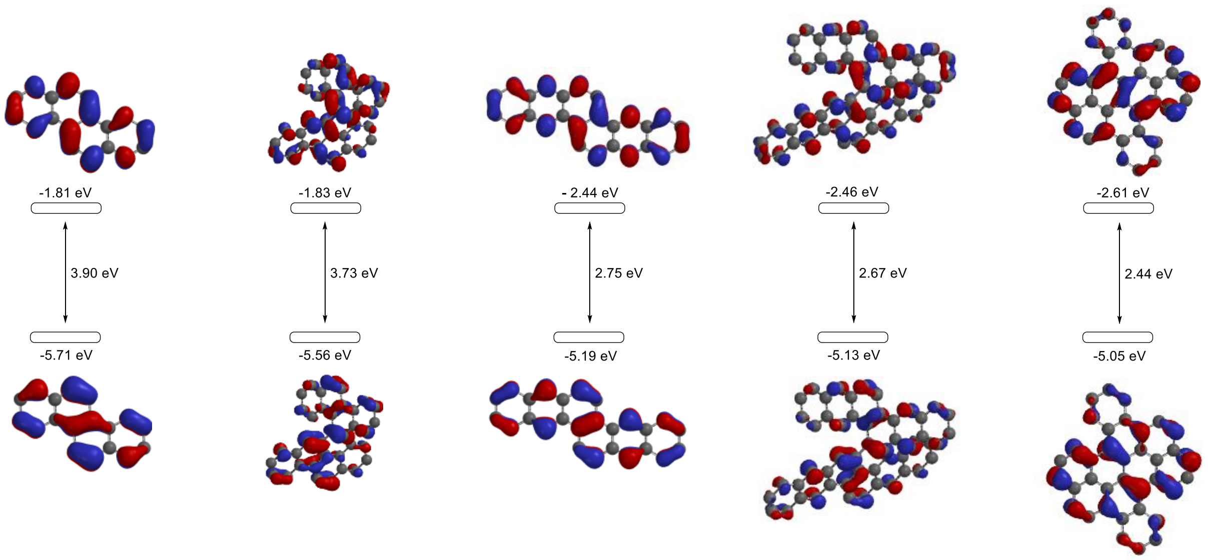

Figure S14: Theoretical gap was obtained from quantum-mechanical calculations with gaussian16 B3LYP/def2SVP // gaussian16 B3LYP/def2TZVP.

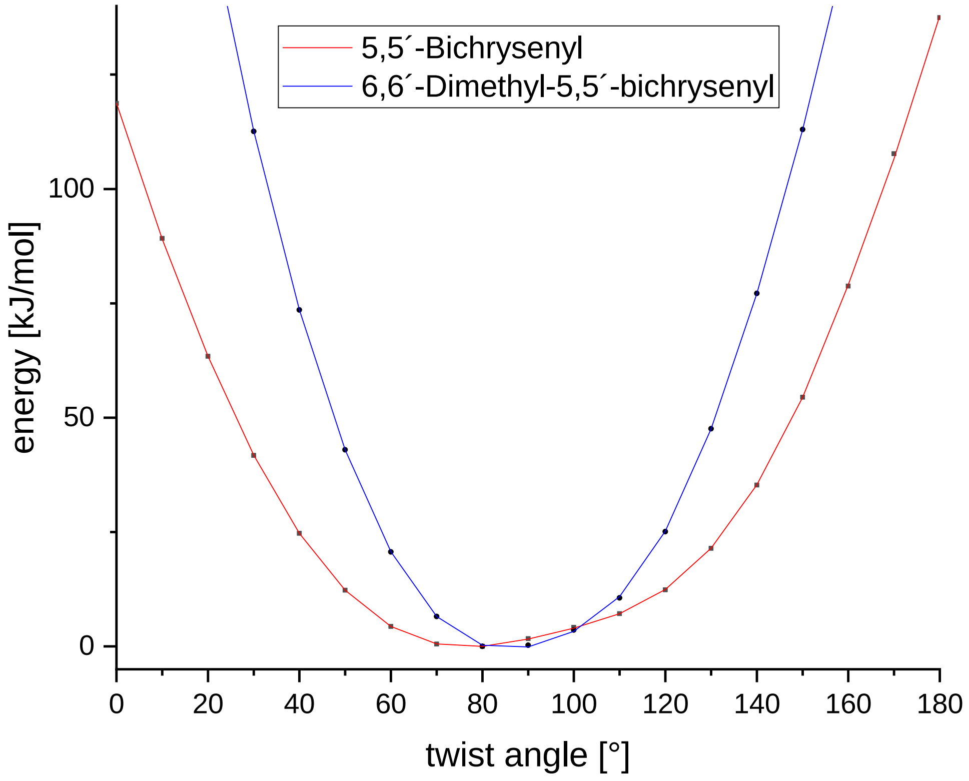

Figure S15: *In silico* analysis of the rotamers of 5,5'-bichrysenyl and 6,6'-dimethyl-5,5'-bichrysenyl by stepwise changing the torsional angle (DFT, B3LYP/6-311++G**, gas phase).

S9. NMR spectra

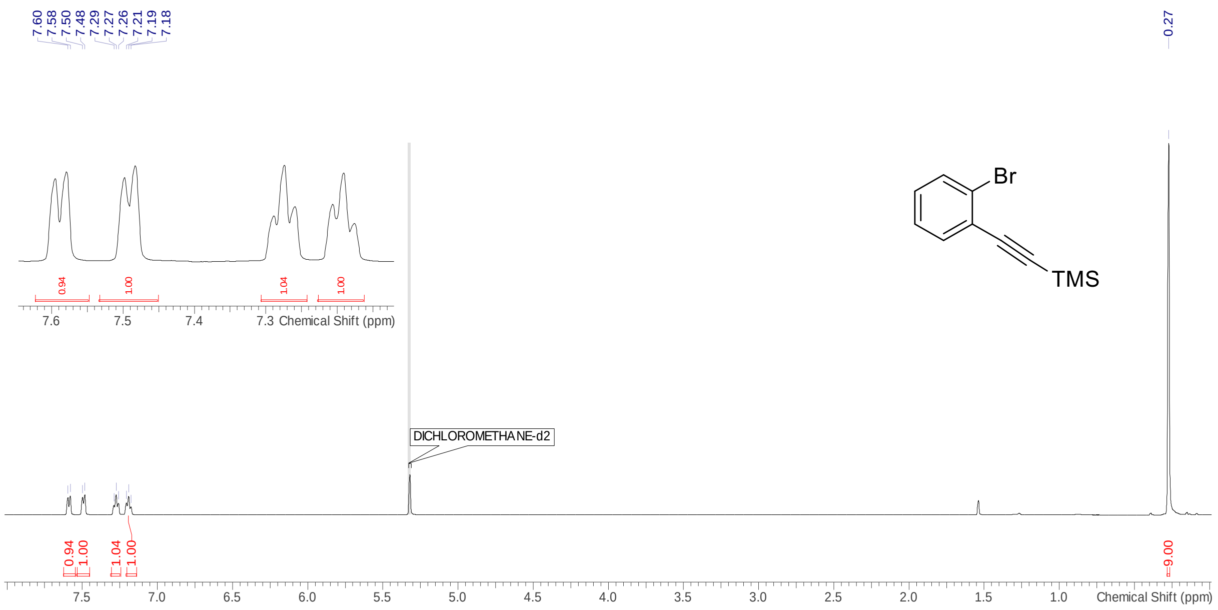

Figure S16: ^{1}H NMR spectrum (500 MHz, 295 K) of **S2** in CD_2Cl_2.

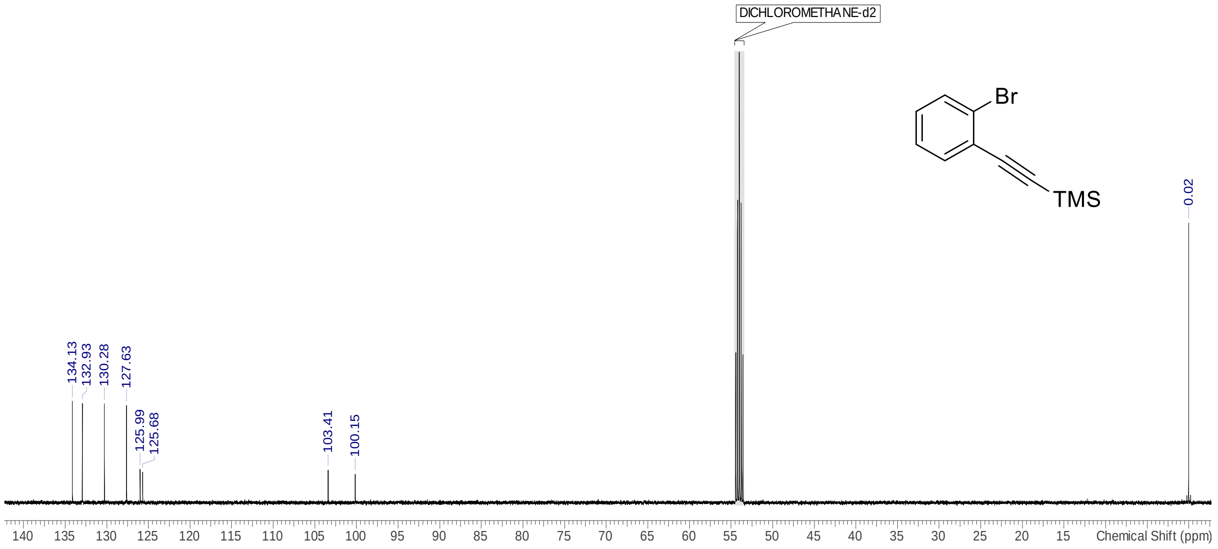

Figure S17: ^{13}C NMR spectrum (126 MHz, 295 K) of **S2** in CD_2Cl_2.

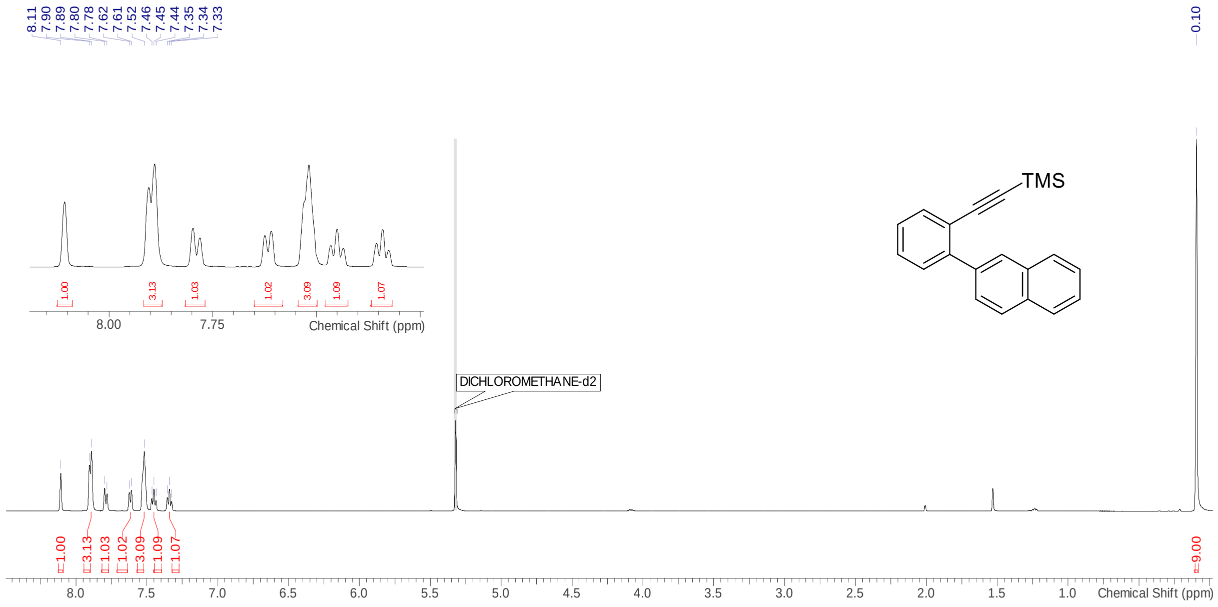

Figure S18: ^{1}H NMR spectrum (500 MHz, 295 K) of **1** in CD_2Cl_2.

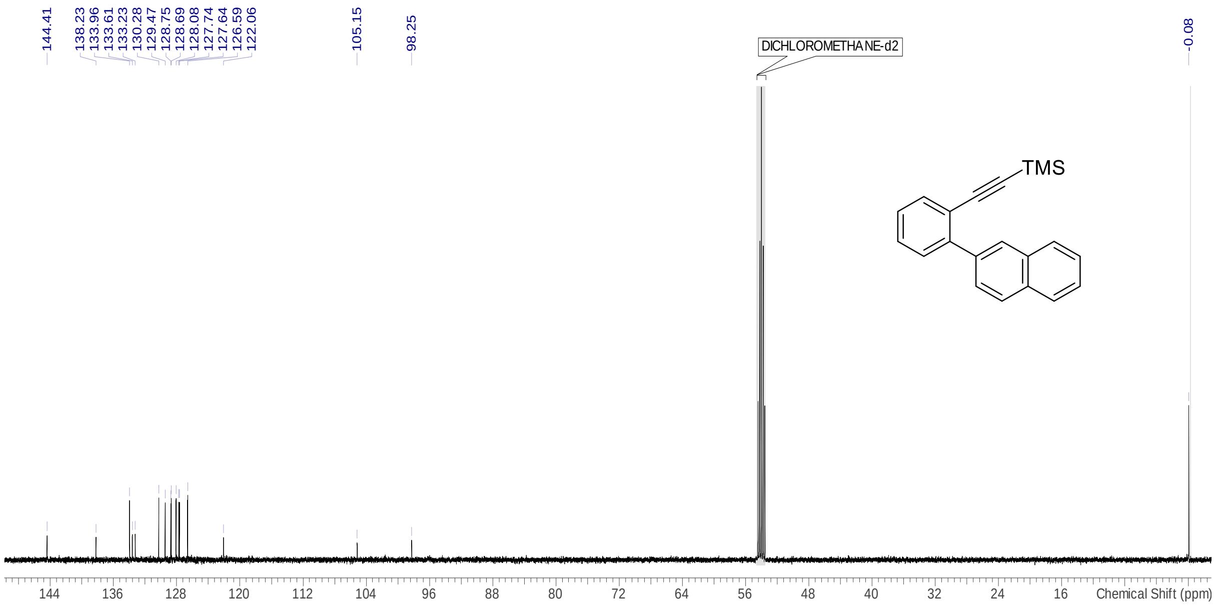

Figure S19: ^{13}C NMR spectrum (126 MHz, 295 K) of **1** in CD_2Cl_2.

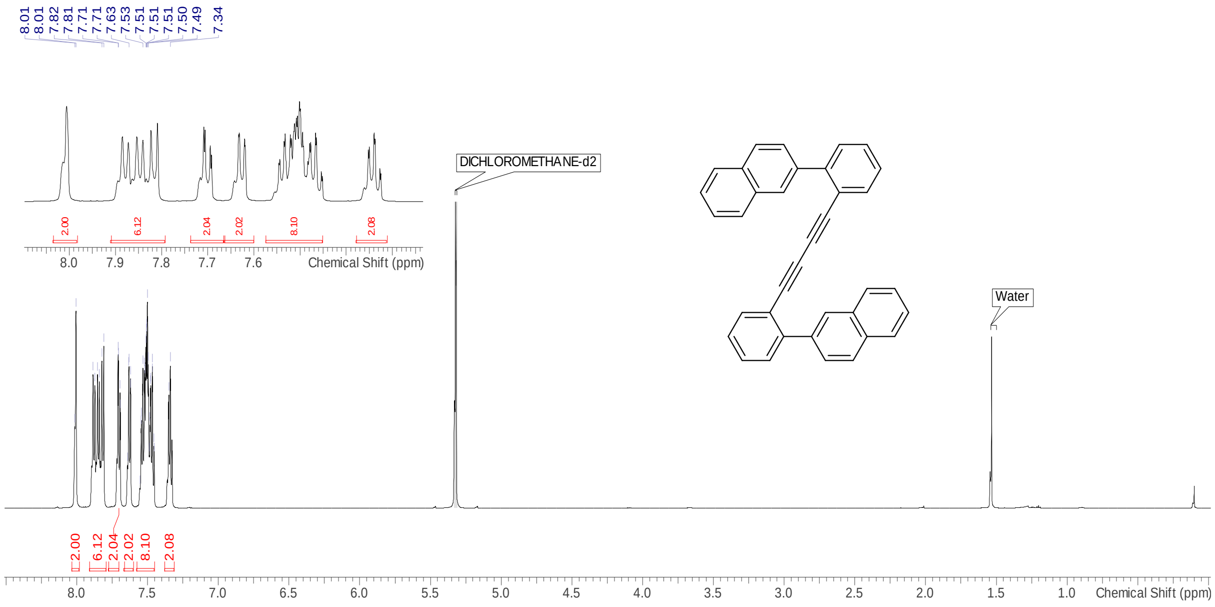

Figure S20: ^{1}H NMR spectrum (600 MHz, 295 K) of **4** in CD_2Cl_2.

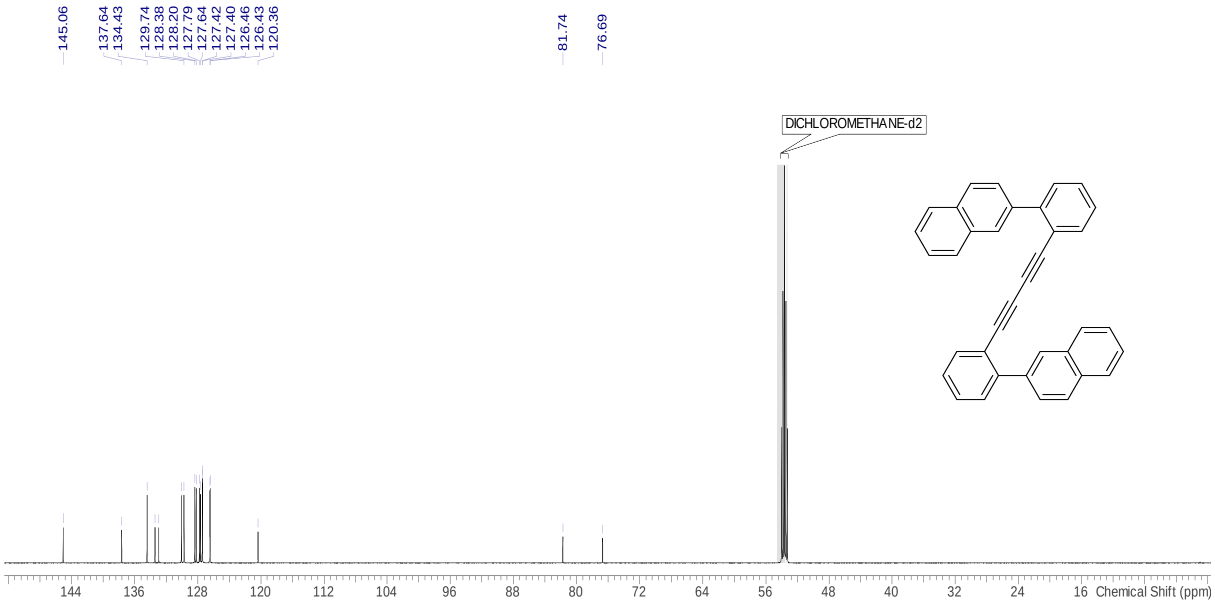

Figure S21: ^{13}C NMR spectrum (151 MHz, 295 K) of **4** in CD_2Cl_2.

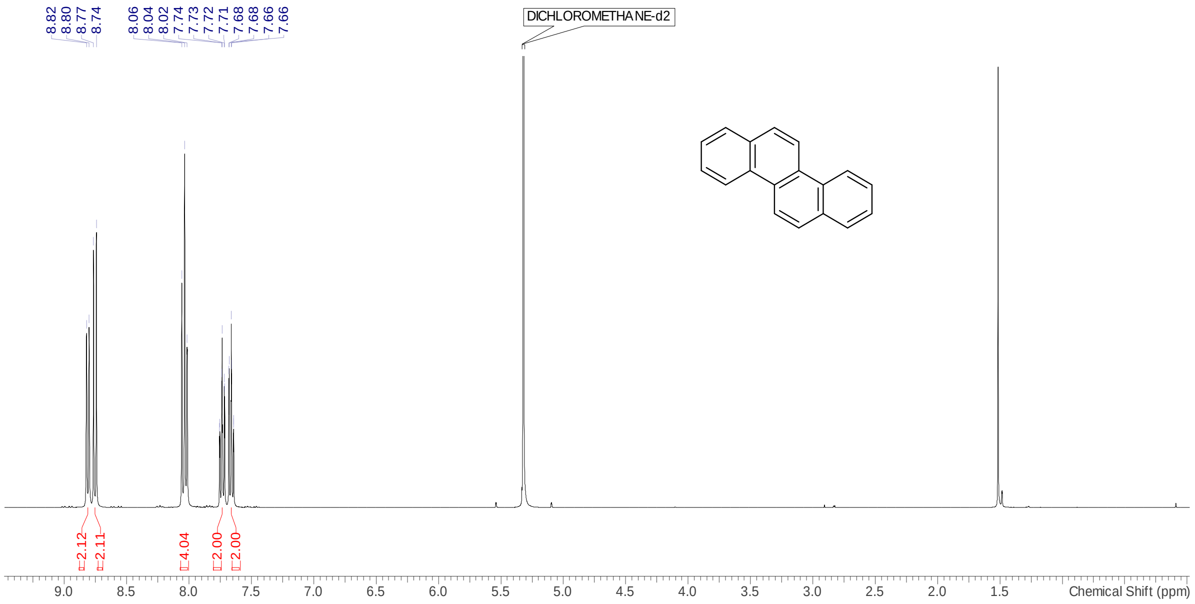

Figure S22: ^{1}H NMR spectrum (400 MHz, 295 K) of **3** in CD_2Cl_2.

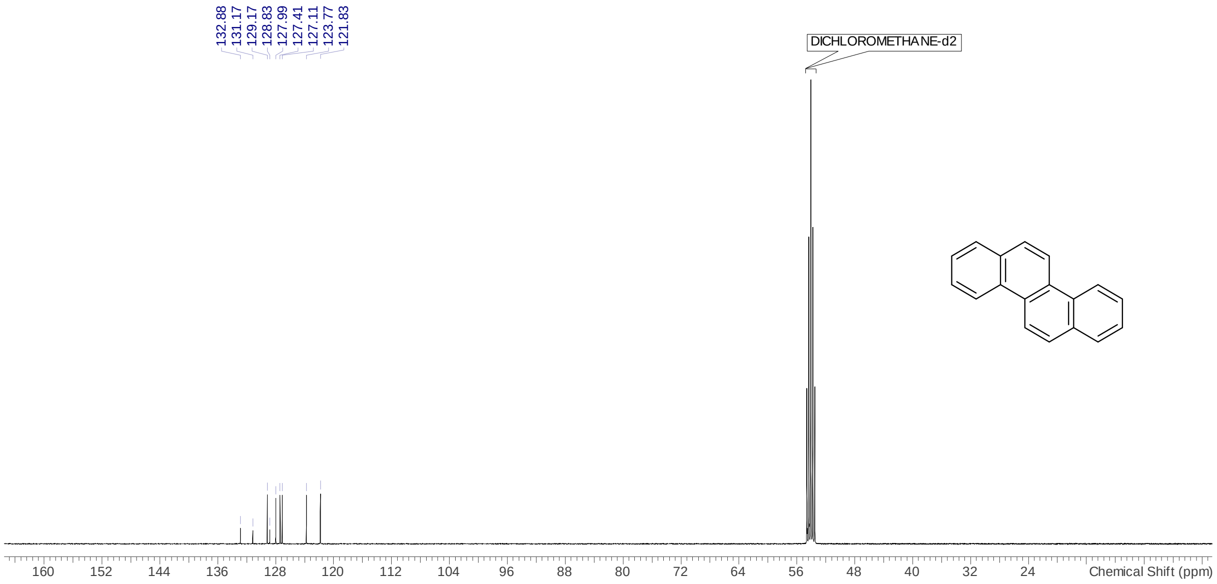

Figure S23: ^{13}C NMR spectrum (101 MHz, 295 K) of **3** in CD_2Cl_2.

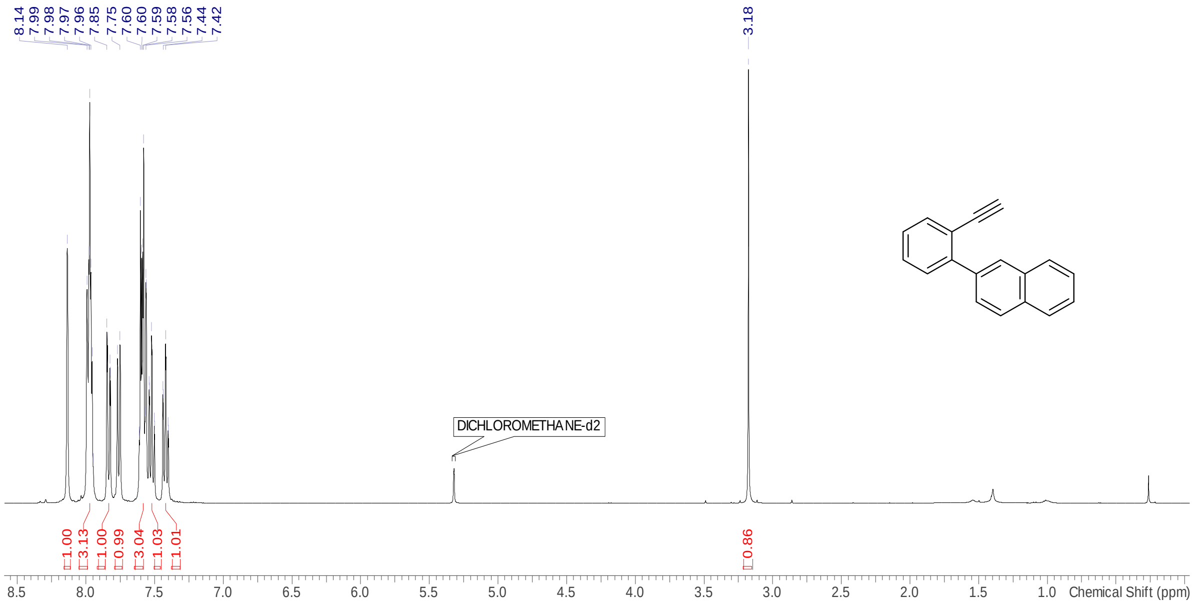

Figure S24: ^{1}H NMR spectrum (400 MHz, 295 K) of **2** in CD_2Cl_2.

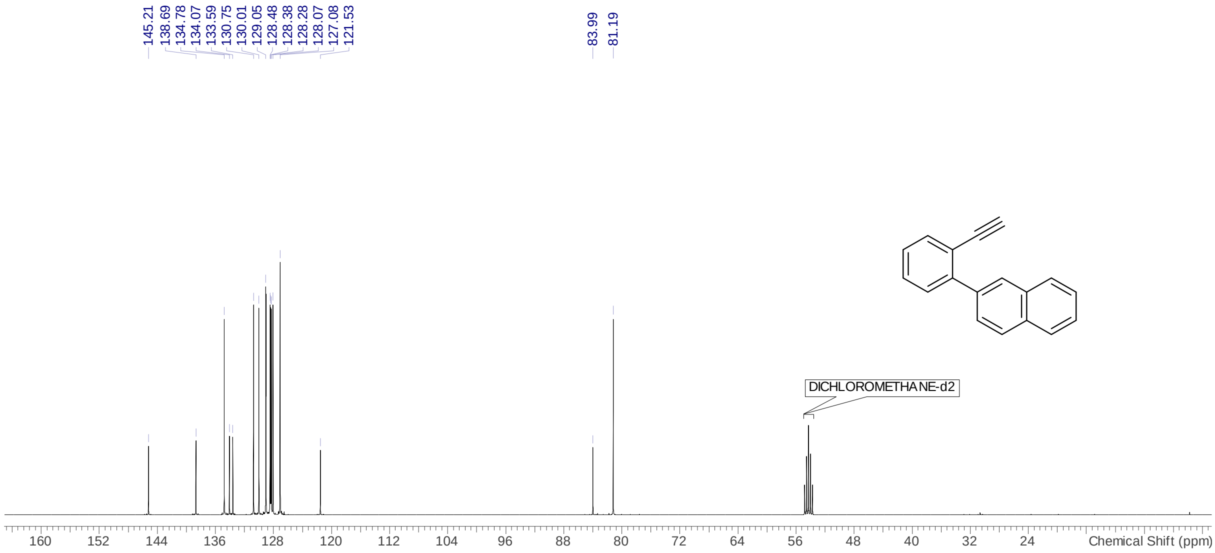

Figure S25: ^{13}C NMR spectrum (101 MHz, 295 K) of **2** in CD_2Cl_2.

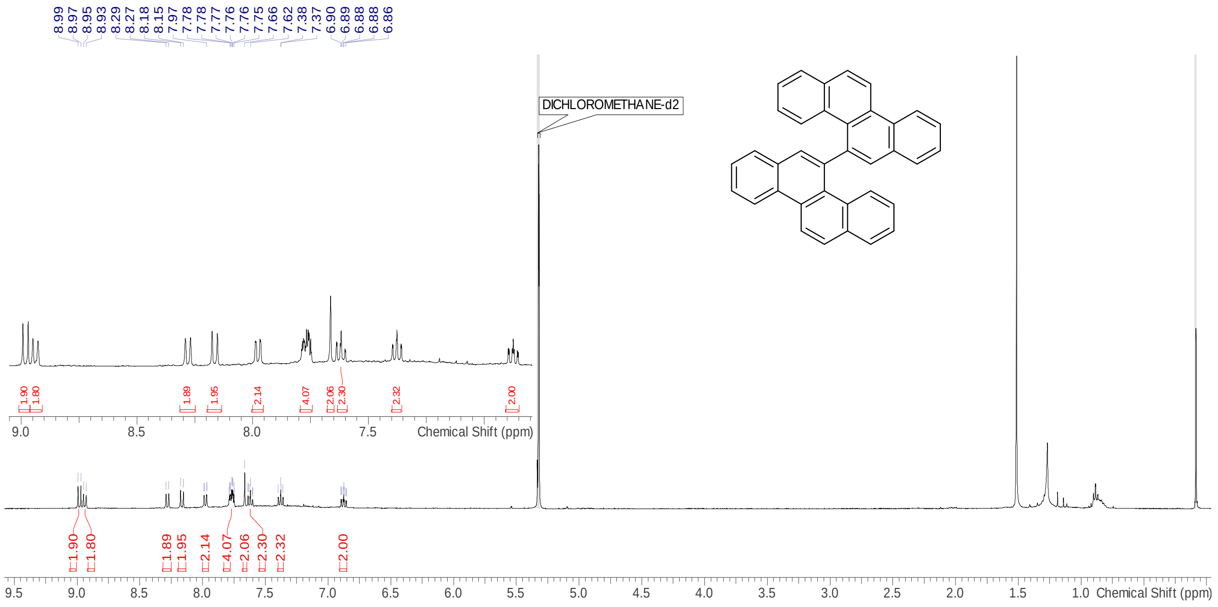

Figure S26: ^1H NMR spectrum (600 MHz, 295 K) of **5** in CD_2Cl_2.

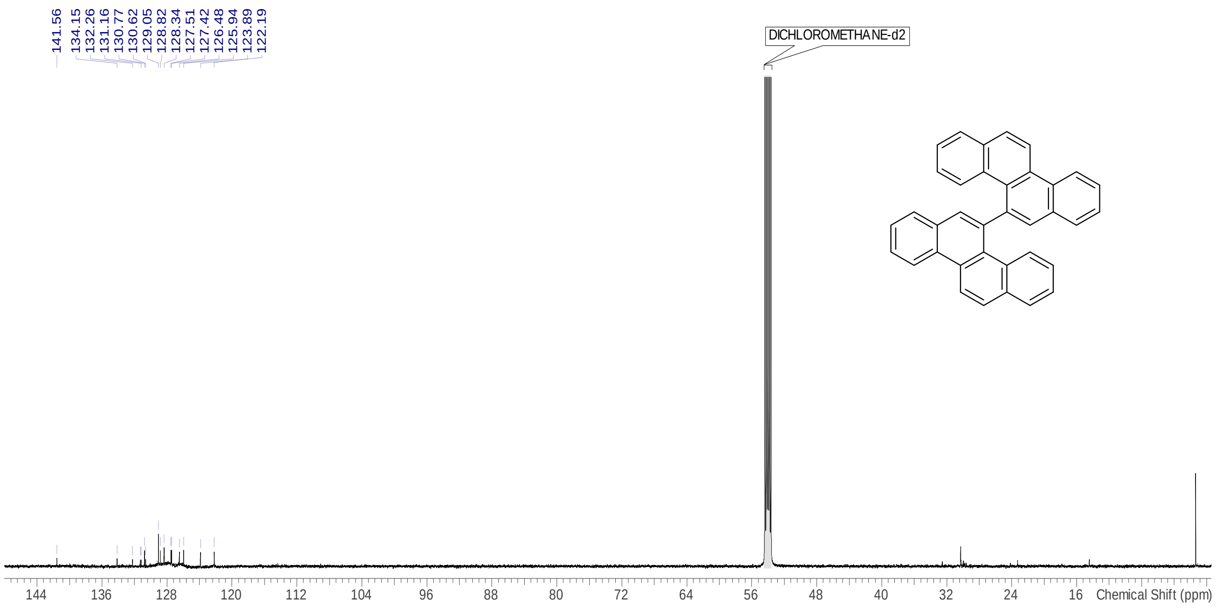

Figure S27: ^{13}C NMR spectrum (151 MHz, 295 K) of **5** in CD_2Cl_2.

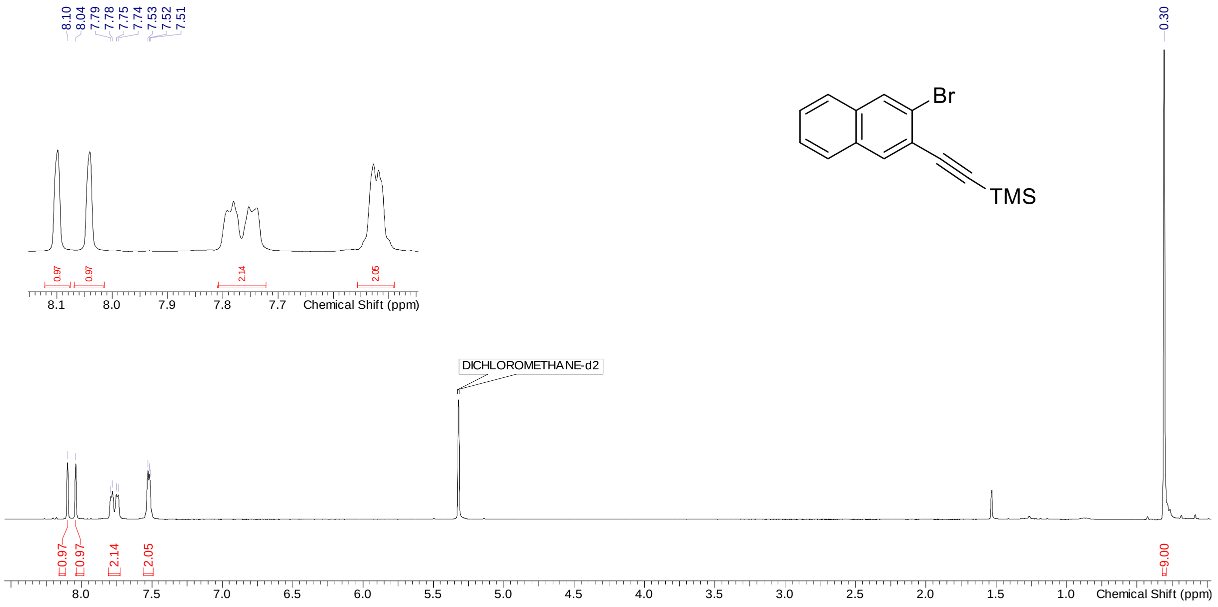

Figure S28: ^{1}H NMR spectrum (500 MHz, 295 K) of **7** in CD_2Cl_2.

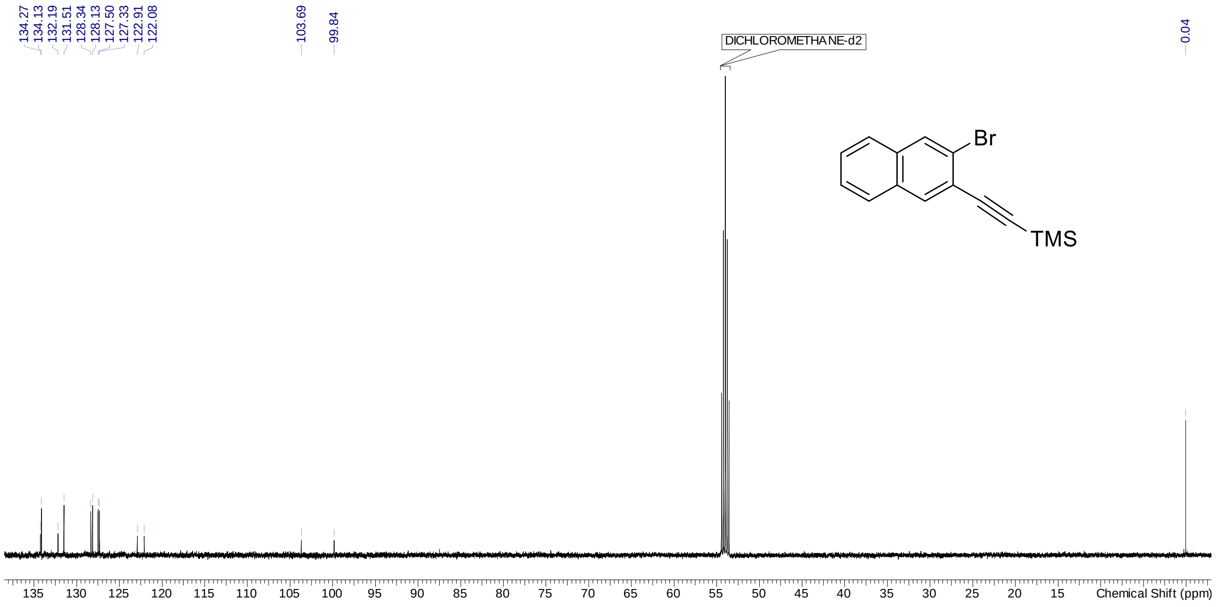

Figure S29: ^{13}C NMR spectrum (126 MHz, 295 K) of **7** in CD_2Cl_2.

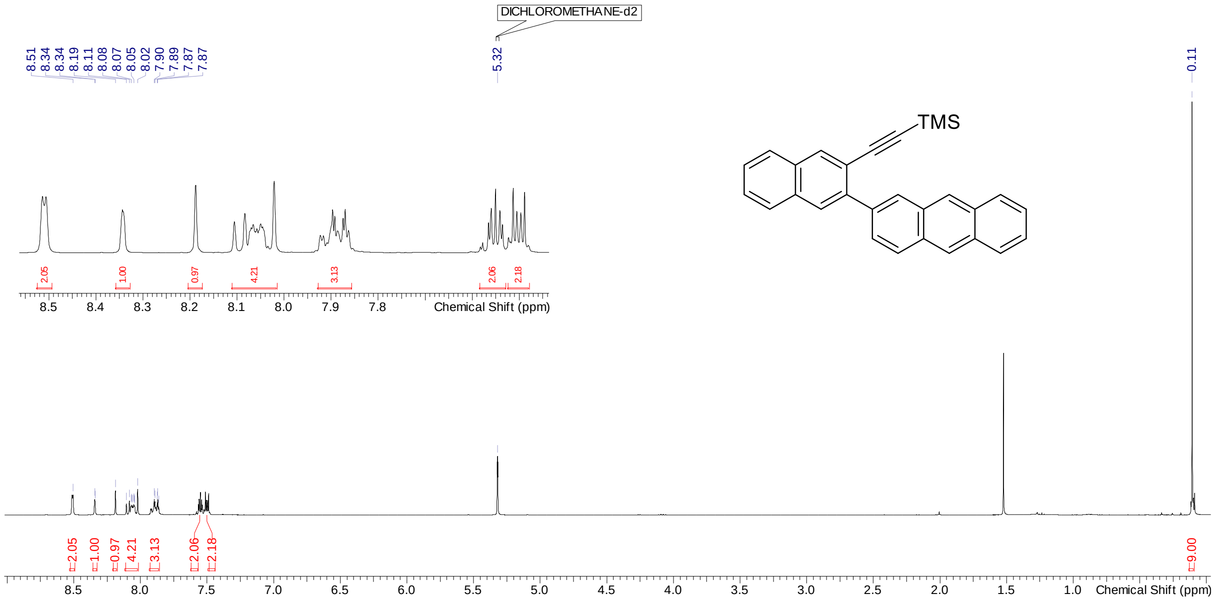

Figure S30: ^{1}H NMR spectrum (400 MHz, 295 K) of **9** in CD_2Cl_2.

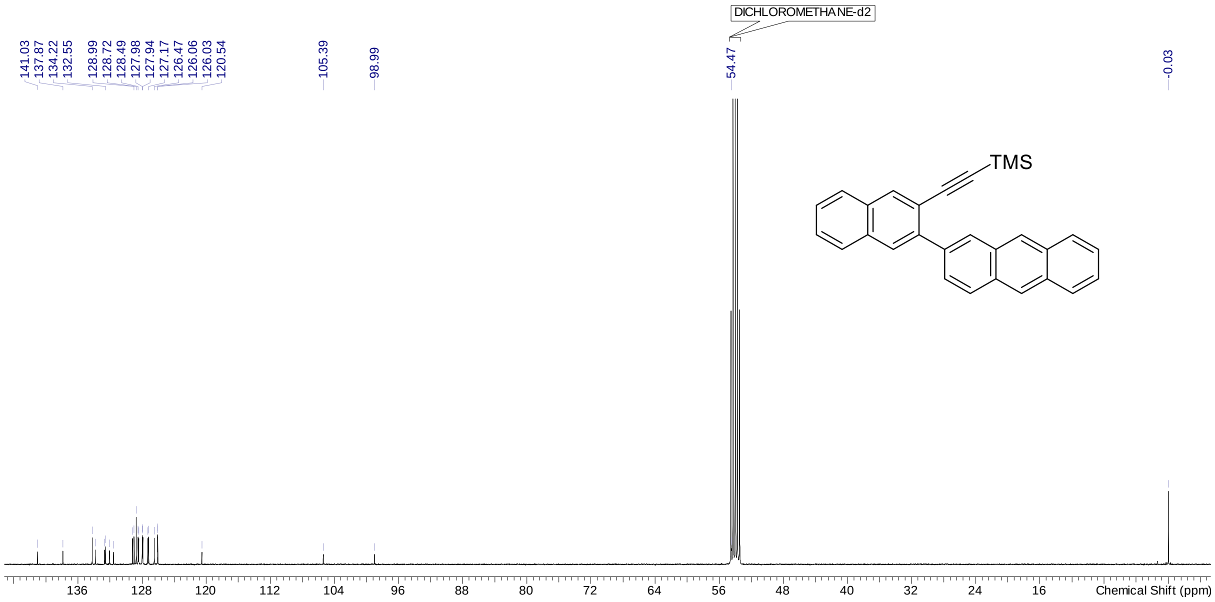

Figure S31: ^{13}C NMR spectrum (101 MHz, 295 K) of **9** in CD_2Cl_2.

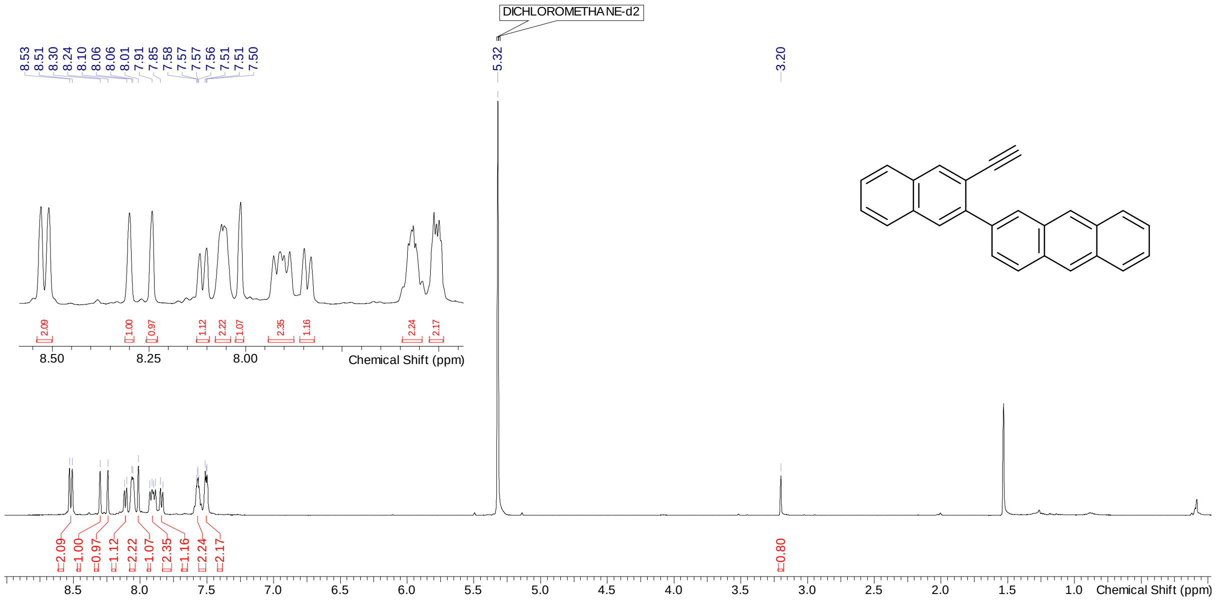

Figure S32: ^{1}H NMR spectrum (500 MHz, 295 K) of **10** in CD_2Cl_2.

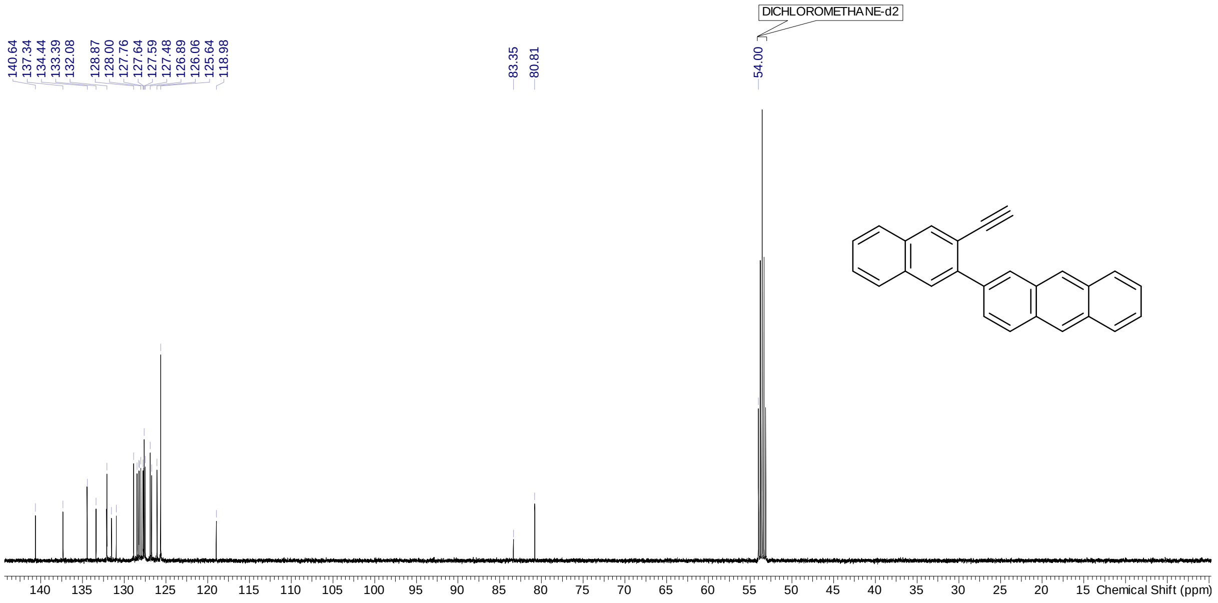

Figure S33: ^{13}C NMR spectrum (126 MHz, 295 K) of **10** in CD_2Cl_2.

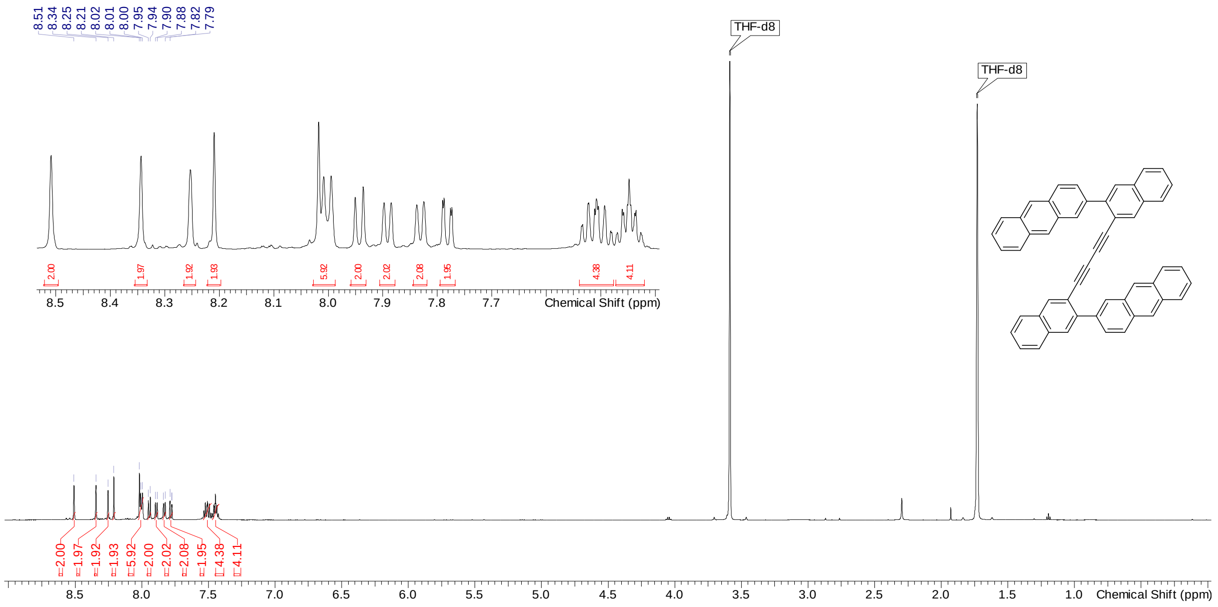

Figure S34: ^{1}H NMR spectrum (600 MHz, 328 K) of **12** in THF-d_8.

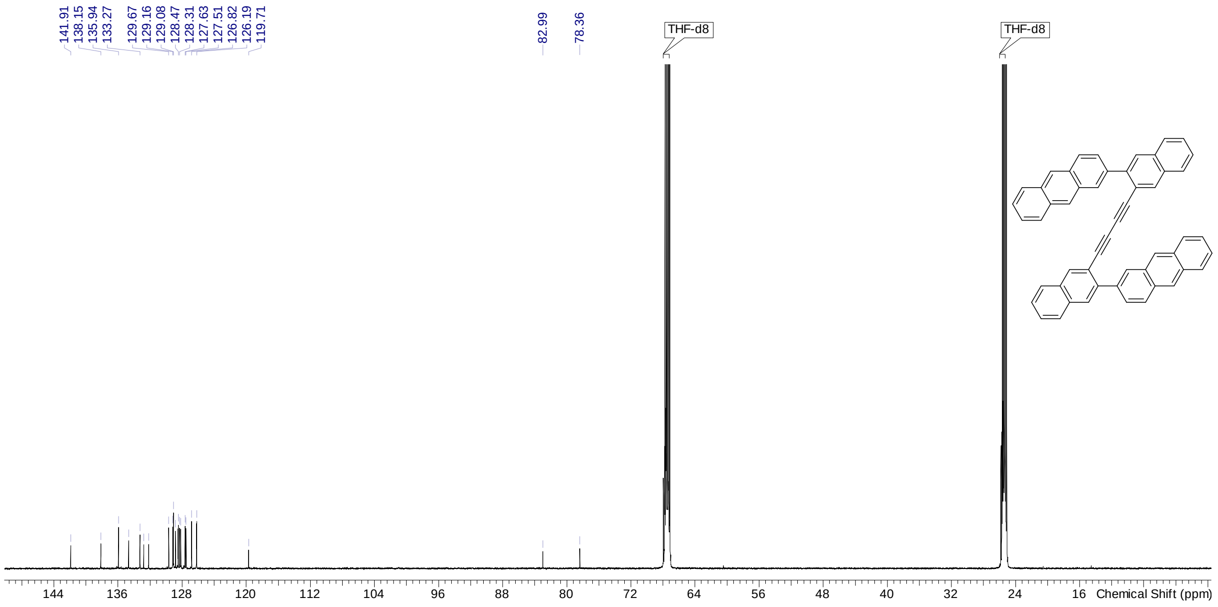

Figure S35: ^{13}C NMR spectrum (151 MHz, 328 K) of **12** in THF-d_8.

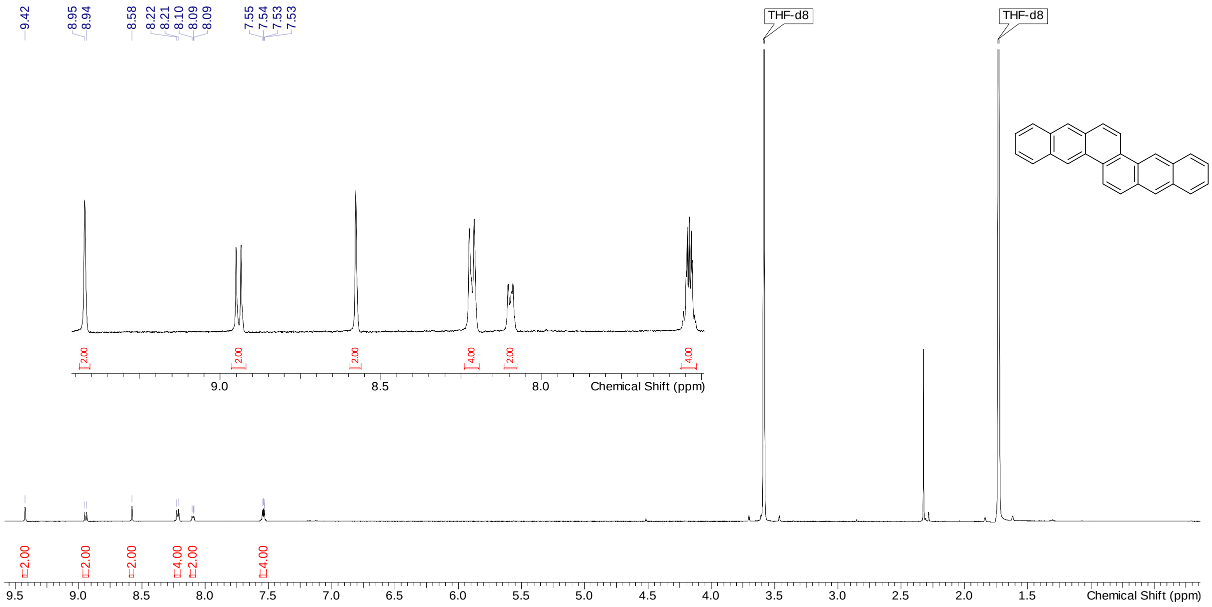

Figure S36: ^{1}H NMR spectrum (600 MHz, 323 K) of **11** in THF-d_8.

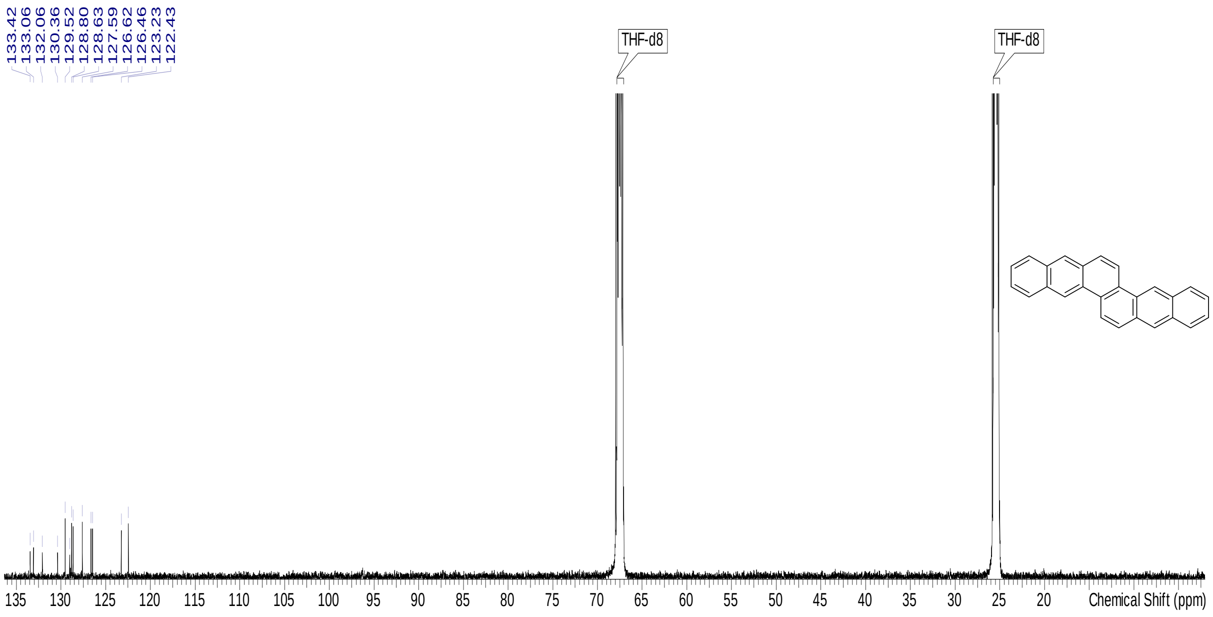

Figure S37: ^{13}C{^{1}H} NMR spectrum (151 MHz, 323 K) of **11** in THF-d_8.

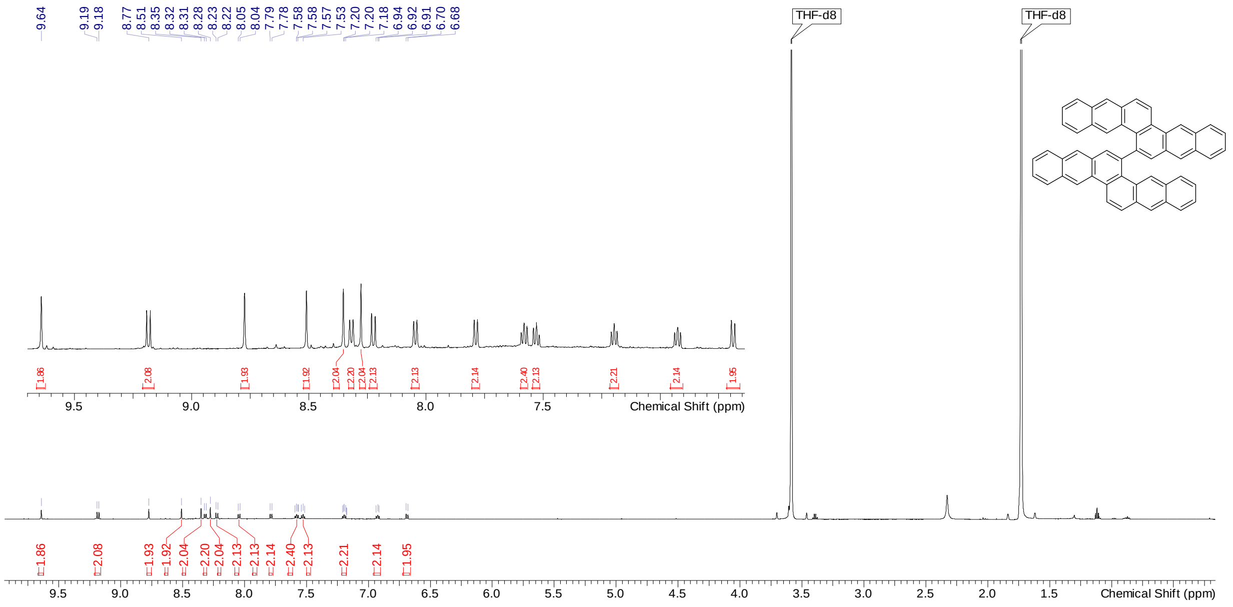

Figure S38: ^{1}H NMR spectrum (600 MHz, 323 K) of **13** in THF-d_8.

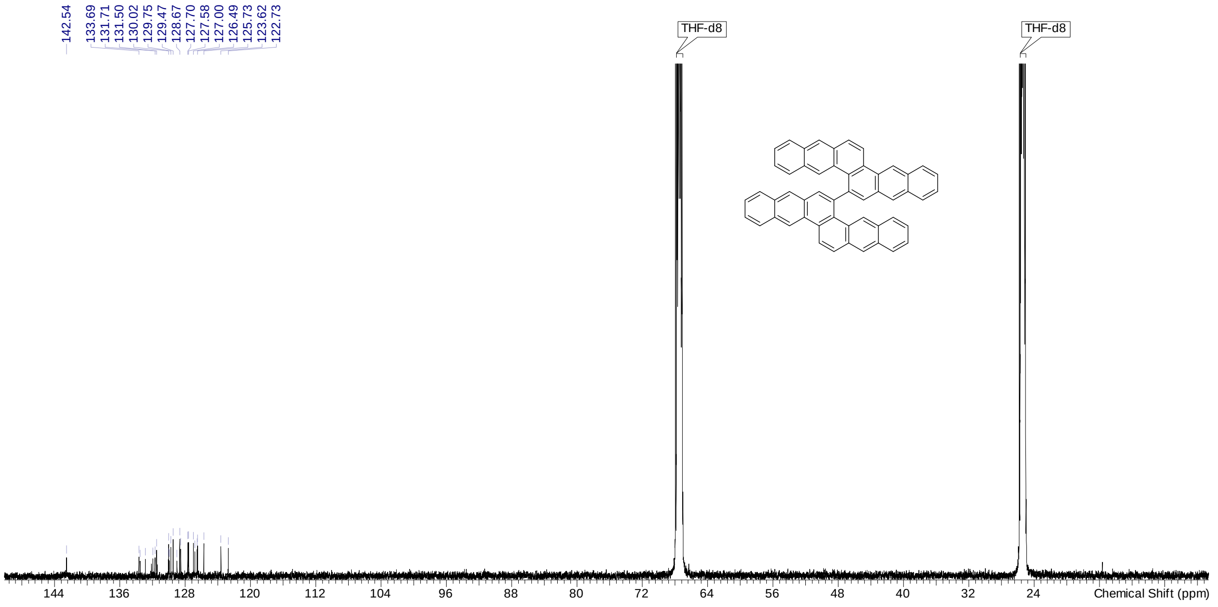

Figure S39: $^{13}C\{^{1}H\}$ NMR spectrum (151 MHz, 323 K) of **13** in THF-d_8.

S10. Crystallographic data

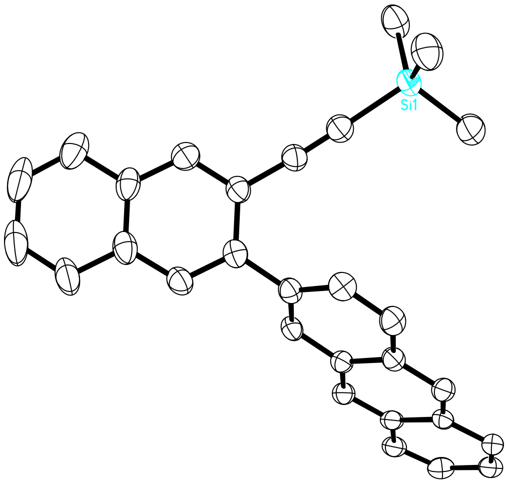

Figure S40: ORTEP structure of **9**.

Empirical formula	$C_{29}H_{24}Si$	
Formula weight	400.57	
Temperature	200(2) K	
Wavelength	0.71073 Å	
Crystal system	monoclinic	
Space group	$P2_1/n$	
Z	4	
Unit cell dimensions	a = 18.8486(5) Å	α = 90 deg.
	b =6.0974(2) Å	β = 102.615(2) deg.
	c = 20.0151(5) Å	γ = 90 deg.
Volume	2244.76(11) $Å^3$	
Density (calculated)	1.18 g/cm^3	
Absorption coefficient	0.12 mm^{-1}	
Crystal shape	needle	
Crystal size	0.233 x 0.032 x 0.027 mm^3	
Crystal colour	colourless	
Theta range for data collection	1.3 to 26.9 deg.	
Index ranges	-23h≤23, -7≤k≤7, -25≤l≤25	
Reflections collected	29990	
Independent reflections	4867 (R(int) = 0.1081)	
Observed reflections	2899 (I > 2σ(I))	
Absorption correction	Semi-empirical from equivalents	
Max. and min. transmission	0.96 and 0.91	
Refinement method	Full-matrix least-squares on F^2	
Data/restraints/parameters	4867 / 0 / 274	
Goodness-of-fit on F^2	1.00	
Final R indices (I>2sigma(I))	R1 = 0.054, wR2 = 0.111	
Largest diff. peak and hole	0.18 and -0.26 $eÅ^{-3}$	

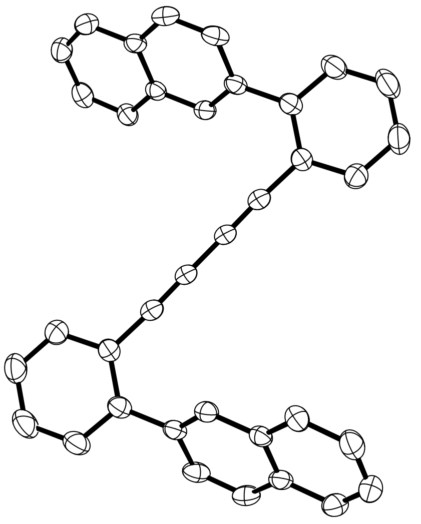

Figure S41: ORTEP structure of **4**.

Empirical formula	$C_{36}H_{22}$	
Formula weight	454.53	
Temperature	200(2) K	
Wavelength	0.71073 Å	
Crystal system	triclinic	
Space group	$P\bar{1}$	
Z	2	
Unit cell dimensions	a =9.9864(6) Å	α = 68.1830(12) deg.
	b = 11.3259(7) Å	β = 87.1386(13) deg.
	c = 12.0789(7) Å	γ = 72.4982(12) deg.
Volume	1206.66(13) $Å^3$	
Density (calculated)	1.25 g/cm^3	
Absorption coefficient	0.07 mm^{-1}	
Crystal shape	plank	
Crystal size	0.200 x 0.140 x 0.065 mm^3	
Crystal colour	colourless	
Theta range for data collection	1.8 to 26.1 deg.	
Index ranges	$-12 \le h \le 12$, $-14 \le k \le 14$, $-14 \le l \le 14$	
Reflections collected	16602	
Independent reflections	4788 (R(int) = 0.0351)	
Observed reflections	3499 (I > 2σ(I))	
Absorption correction	Semi-empirical from equivalents	
Max. and min. transmission	0.96 and 0.93	
Refinement method	Full-matrix least-squares on F^2	
Data/restraints/parameters	4788 / 0 / 325	
Goodness-of-fit on F^2	1.03	
Final R indices (I>2sigma(I))	R1 = 0.045, wR2 = 0.104	
Largest diff. peak and hole	0.15 and -0.19 $eÅ^{-3}$	

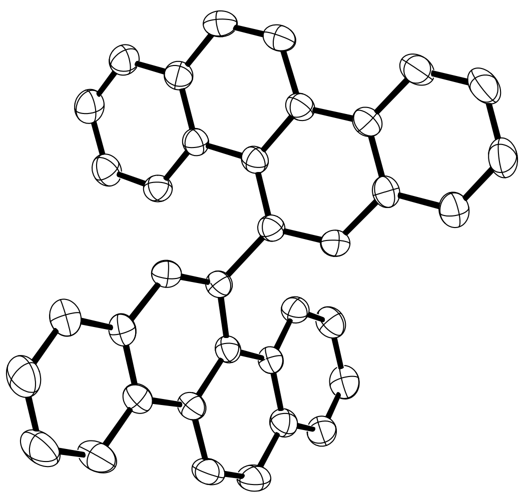

Figure S42: ORTEP structure of **5**.

Summenformel	$C_{36}H_{22}$	
Molmasse	454.53	
Temperatur	200(2) K	
Wellenlänge	1.54178 Å	
Kristallsystem	tetragonal	
Raumgruppe	$P4_32_12$	
Z	4	
Gitterkonstanten	a =8.9409(4) Å	α = 90 °
	b =8.9409(4) Å	β = 90 °
	c = 28.696(3) Å	γ = 90 °
Zellvolumen	2293.9(3) $Å^3$	
Dichte (berechnet)	1.316 g/cm^3	
Absorptionskoeffizient µ	0.567 mm^{-1}	
Kristallform	brick	
Kristallgröße	0.100 x 0.040 x 0.027 mm^3	
Kristallfarbe	pale yellow	
Gemessener Theta-Bereich	5.181 bis 66.556 °	
Indexgrenzen	-4≤h≤10, -10≤k≤8, -34≤l≤33	
Gemessene Reflexe	8186	
Unabhängige Reflexe	2020 (R(int) = 0.0457)	
Beobachtete Reflexe	1510 (I > 2σ(I))	
Absorptionskorrektur	Semi-empirical from equivalents	
Max/min Transmission	1.68 and 0.61	
Strukturverfeinerung	Full-matrix least-squares an F^2	
Daten/Restraints/Parameter	2020 / 0 / 163	
Goodness-of-fit an F^2	1.05	
R-Werte (I>2sigma(I))	R1 = 0.045, wR2 = 0.085	
Flack-Parameter	-0.2(10)	
Extinktionskoeffizient	n/a	
Max/min Restelektronendichte	0.14 und -0.14 $eÅ^{-3}$	

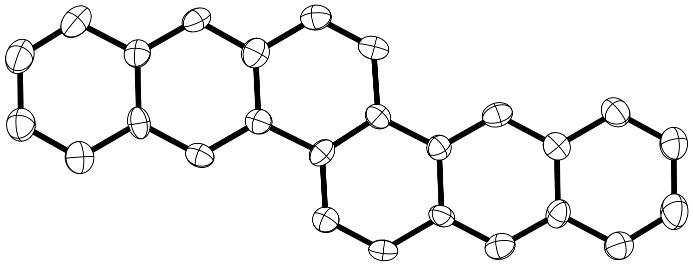

Figure S43: ORTEP structure of **11**.

Empirical formula	$C_{26}H_{16}$	
Formula weight	328.39	
Temperature	200(2) K	
Wavelength	1.54178 Å	
Crystal system	monoclinic	
Space group	$P2_1$	
Z	2	
Unit cell dimensions	a =6.6158(10) Å	α = 90 deg.
	b =7.4748(8) Å	β = 91.142(12) deg.
	c = 16.296(2) Å	γ = 90 deg.
Volume	805.73(19) $Å^3$	
Density (calculated)	1.35 g/cm^3	
Absorption coefficient	0.58 mm^{-1}	
Crystal shape	needle	
Crystal size	0.090 x 0.024 x 0.020 mm^3	
Crystal colour	yellow	
Theta range for data collection	6.7 to 67.0 deg.	
Index ranges	$-7\leq h\leq 7$, $-8\leq k\leq 7$, $-10\leq l\leq 19$	
Reflections collected	5433	
Independent reflections	2327 (R(int) = 0.0670)	
Observed reflections	1121 (I > 2σ(I))	
Absorption correction	Semi-empirical from equivalents	
Max. and min. transmission	1.51 and 0.69	
Refinement method	Full-matrix least-squares on F^2	
Data/restraints/parameters	2327 / 1 / 235	
Goodness-of-fit on F^2	1.02	
Final R indices (I>2sigma(I))	R1 = 0.054, wR2 = 0.097	
Absolute structure parameter	4.5(10)	
Largest diff. peak and hole	0.19 and -0.25 $eÅ^{-3}$	

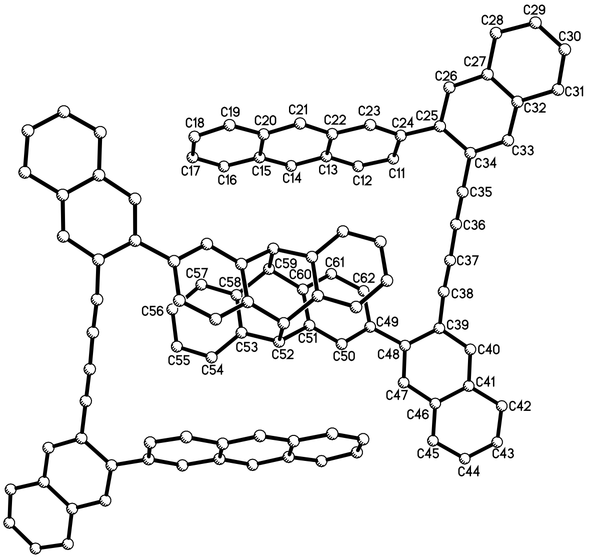

Figure S44: ORTEP structure of **12** as a Dimer.

Empirical formula	$C_{104}H_{60}$	
Formula weight	1309.52	
Temperature	240(2) K	
Wavelength	1.54178 Å	
Crystal system	monoclinic	
Space group	$P2_1/c$	
Z	2	
Unit cell dimensions	a = 21.410(4) Å	α =90 deg.
	b = 25.670(4) Å	β =99.893(14) deg.
	c =7.7044(12) Å	γ =90 deg.
Volume	4171.2(12) $Å^3$	
Density (calculated)	1.04 g/cm^3	
Absorption coefficient	0.45 mm^{-1}	
Crystal shape	plate	
Crystal size	0.090 x 0.040 x 0.010 mm^3	
Crystal colour	pale yellow	
Theta range for data collection	6.1 to 33.4 deg.	
Index ranges	$-15 \le h \le 15$, $-17 \le k \le 18$, $-5 \le l \le 5$	
Reflections collected	6466	
Independent reflections	1587 (R(int) = 0.0826)	
Observed reflections	773 (I I > 2 σ (I))	
Absorption correction	Semi-empirical from equivalents	
Max. and min. transmission	1.60 and 0.72	
Refinement method	Full-matrix least-squares on F^2	
Data/restraints/parameters	1587 / 136 / 209	
Goodness-of-fit on F^2	1.03	
Final R indices (I>2sigma(I))	R1 = 0.085, wR2 = 0.208	
Largest diff. peak and hole	0.19 and -0.15 $eÅ^{-3}$	

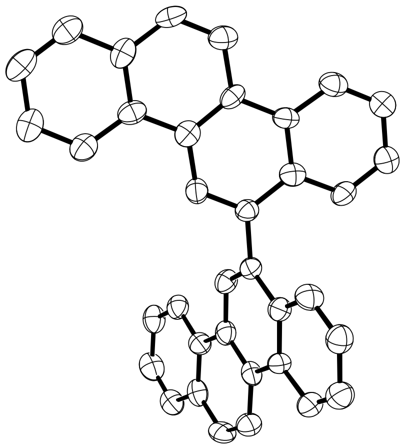

Figure S45: ORTEP structure of 6,6´-bichrysenyl.

Empirical formula	$C_{36}H_{22}$	
Formula weight	454.53	
Temperature	200(2) K	
Wavelength	1.54178 Å	
Crystal system	monoclinic	
Space group	C2/c	
Z 4		
Unit cell dimensions	a = 25.447(3) Å	α =90 deg.
	b =6.3577(8) Å	β =114.900(8) deg.
	c = 15.1015(17) Å	γ =90 deg.
Volume	2216.1(5) $Å^3$	
Density (calculated)	1.36 g/cm^3	
Absorption coefficient	0.59 mm^{-1}	
Crystal shape	plate	
Crystal size	0.058 x 0.046 x 0.015 mm^3	
Crystal colour	colourless	
Theta range for data collection	3.8 to 54.2 deg.	
Index ranges	$-26 \leq h \leq 23$, $-6 \leq k \leq 6$, $-12 \leq l \leq 15$	
Reflections collected	6775	
Independent reflections	1356 (R(int) = 0.0906)	
Observed reflections	746 ($I > 2\sigma(I)$)	
Absorption correction	Semi-empirical from equivalents	
Max. and min. transmission	1.75 and 0.65	
Refinement method	Full-matrix least-squares on F^2	
Data/restraints/parameters	1356 / 153 / 163	
Goodness-of-fit on F^2	1.17	
Final R indices (I>2sigma(I))	R1 = 0.087, wR2 = 0.163	
Largest diff. peak and hole	0.26 and -0.22 $eÅ^{-3}$	

S11. References

[1] G. R. Fulmer, A. J. M. Miller, N. H. Sherden, H. E. Gottlieb, A. Nudelman, B. M. Stoltz, J. E. Bercaw, K. I. Goldberg, *Organometallics* **2010**, *29*, 2176-2179.

[2] M. J. Frisch, G. W. Trucks, H. B. Schlegel, G. E. Scuseria, M. A. Robb, J. R. Cheeseman, G. Scalmani, V. Barone, G. A. Petersson, H. Nakatsuji, X. Li, M. Caricato, A. V. Marenich, J. Bloino, B. G. Janesko, R. Gomperts, B. Mennucci, H. P. Hratchian, J. V. Ortiz, A. F. Izmaylov, J. L. Sonnenberg, Williams, F. Ding, F. Lipparini, F. Egidi, J. Goings, B. Peng, A. Petrone, T. Henderson, D. Ranasinghe, V. G. Zakrzewski, J. Gao, N. Rega, G. Zheng, W. Liang, M. Hada, M. Ehara, K. Toyota, R. Fukuda, J. Hasegawa, M. Ishida, T. Nakajima, Y. Honda, O. Kitao, H. Nakai, T. Vreven, K. Throssell, J. A. Montgomery Jr., J. E. Peralta, F. Ogliaro, M. J. Bearpark, J. J. Heyd, E. N. Brothers, K. N. Kudin, V. N. Staroverov, T. A. Keith, R. Kobayashi, J. Normand, K. Raghavachari, A. P. Rendell, J. C. Burant, S. S. Iyengar, J. Tomasi, M. Cossi, J. M. Millam, M. Klene, C. Adamo, R. Cammi, J. W. Ochterski, R. L. Martin, K. Morokuma, O. Farkas, J. B. Foresman, D. J. Fox, Wallingford, CT, **2016**.

[3] C. M. Cardona, W. Li, A. E. Kaifer, D. Stockdale, G. C. Bazan, *Adv. Mater.* **2011**, *23*, 2367-2371.

[4] S. B. Lee, K. H. Park, C. W. Joo, J.-I. Lee, J. Lee, Y.-H. Kim, *Dyes Pigments* **2016**, *128*, 19-25.

[5] F. Cottet, E. Castagnetti, M. Schlosser, *Synth.* **2005**, 798-803.

[6] S. Rondeau-Gagné, C. Curutchet, F. Grenier, G. D. Scholes, J.-F. Morin, *Tetrahedron* **2010**, *66*, 4230-4242.

[7] T.-L. Wu, H.-H. Chou, P.-Y. Huang, C.-H. Cheng, R.-S. Liu, *J. Org. Chem.* **2014**, *79*, 267-274.

[8] R. E. Messersmith, M. A. Siegler, J. D. Tovar, *Synlett* **2018**, *29*, 2499-2502.

[9] Crystallographic data from the Cambridge Structural Database (CCDC: 1483901)
